# RNAi phenotype profiling of kinases identifies potential therapeutic targets in Ewing's sarcoma

**DOI:** 10.1186/1476-4598-9-218

**Published:** 2010-08-18

**Authors:** Shilpi Arora, Irma M Gonzales, R Tanner Hagelstrom, Christian Beaudry, Ashish Choudhary, Chao Sima, Raoul Tibes, Spyro Mousses, David O Azorsa

**Affiliations:** 1 Pharmaceutical Genomic Division, Translational Genomics Research Institute, Scottsdale, AZ, 85259, USA; 2 Cancer and Cell Biology Division, Translational Genomics Research Institute, Phoenix, AZ, 85004, USA; 3 Clinical Translational Research Division, The Translational Genomics Research Institute, Scottsdale, Arizona 85259, USA; 4 Computational Biology Division, Translational Genomics Research Institute, Phoenix, AZ, 85004, USA

## Abstract

**Background:**

Ewing's sarcomas are aggressive musculoskeletal tumors occurring most frequently in the long and flat bones as a solitary lesion mostly during the teen-age years of life. With current treatments, significant number of patients relapse and survival is poor for those with metastatic disease. As part of novel target discovery in Ewing's sarcoma, we applied RNAi mediated phenotypic profiling to identify kinase targets involved in growth and survival of Ewing's sarcoma cells.

**Results:**

Four Ewing's sarcoma cell lines TC-32, TC-71, SK-ES-1 and RD-ES were tested in high throughput-RNAi screens using a siRNA library targeting 572 kinases. Knockdown of 25 siRNAs reduced the growth of all four Ewing's sarcoma cell lines in replicate screens. Of these, 16 siRNA were specific and reduced proliferation of Ewing's sarcoma cells as compared to normal fibroblasts. Secondary validation and preliminary mechanistic studies highlighted the kinases STK10 and TNK2 as having important roles in growth and survival of Ewing's sarcoma cells. Furthermore, knockdown of STK10 and TNK2 by siRNA showed increased apoptosis.

**Conclusion:**

In summary, RNAi-based phenotypic profiling proved to be a powerful gene target discovery strategy, leading to successful identification and validation of STK10 and TNK2 as two novel potential therapeutic targets for Ewing's sarcoma.

## Introduction

Ewing's sarcoma represents approximately three percent of pediatric cancers and is the second most common bone malignancy in children and adolescents [1,2]. It is an aggressive cancer with a tendency to recur following resection and it metastasizes to the lung, bone and bone marrow. Ewing's sarcomas harbor unique chromosomal translocations that give rise to fusion genes that act as oncoproteins [3]. Rearrangement of the EWS gene on chromosome 22q12 with an ETS gene family member is the underlying molecular genetic abnormality for Ewing's sarcoma. The most common translocation involves the genes EWS and Friend Leukemia Integration Site 1 (FLI1). This translocation can be further subdivided into two separate types, Type I and Type II, with Type I resulting from the translocation fusing EWS exon 7 to FLI-1 exon 6 and Type II resulting from the fusion of EWS exon 7 to FLI1 exon 5. The newly formed EWS-FLI1 fusion protein is a transcription factor that can then lead to aberrant transcription [4].

Morphologically, Ewing's sarcoma is composed of small round cells with high nuclear to cytoplasmic ratio and cells from more than 90% of patients express the adhesion receptor CD99 [5,6]. Disease management for patients with localized disease has substantially improved but the prognosis for those with metastatic or recurrent disease has changed very little over the past three decades. Currently, Ewing's sarcoma patients are treated with a combination of surgery, radiation and chemotherapy [7]. Five-year event free survival for patients with metastatic disease is only 20% and curative therapy does not exist for patients whose disease recurs rapidly following therapy for localized disease [7,8]. Recently, expression of several individual genes has been linked to the development and progression of the disease, but so far there has been no comprehensive systematic study undertaken to identify functionally relevant genes in Ewing's sarcoma [9-13]. The genomic translocations in Ewing's sarcoma provide a valuable tool for accurate diagnosis. In addition, these common genetic abnormalities could serve in identifying specific genetic vulnerabilities, which would be useful in development of targeted therapeutics for this disease.

In order to identify novel therapeutic targets for Ewing's sarcoma, we employed a functional genomics approach based on high-throughput RNA interference (HT-RNAi), which is also known as "loss-of-function" screening. The basis of this technology is RNA interference (RNAi), a robust method of post-transcriptional silencing of genes using double-stranded RNA in the form of either siRNA (short interfering RNA) or shRNA (short hairpin RNA) with sequence homology driven specificity [14]. Large-scale libraries of siRNA and shRNA have been used to identify genes involved in many biological functions [15-19]. We utilized a siRNA library targeting human kinases to identify single siRNA kinase targets for Ewing's sarcoma cells. The availability of four Ewing's sarcoma cell lines that transfect well and are amenable to high throughput screening enables us to identify essential kinase that regulate growth of Ewing's sarcoma cells. Numerous small molecule kinase inhibitors to various different targets are fairly well developed and rapid translation of our results into the clinic is a real prospect from such screens. Results from HT-RNAi screening of kinases identified seventeen specific siRNAs (corresponding to sixteen genes) that lead to reduced growth and proliferation of Ewing's sarcoma cells. We showed that two kinases, STK10 and TNK2, are important in survival of Ewing's sarcoma cells and represent potential therapeutic targets for future drug development in this disease.

## Materials and methods

### Cell Culture

The human Ewing's sarcoma cell lines TC-32 and TC-71 were a kind gift from Dr. Javed Khan (Pediatric Oncology Branch, National Cancer Institute, Gaithersburg, MD). The Ewing's sarcoma cell lines RD-ES and SK-ES-1 were obtained from ATCC (Manassas, VA). The human normal fibroblast cell line GM05659 was obtained from the Coriell Institute (Camden, NJ). TC-32, TC-71, and RD-ES cell lines were grown in RPMI, supplemented with 10% FBS, 2 mM L-glutamine, 100 IU/ml penicillin G, and 100 μg/ml streptomycin. SK-ES-1 cells were grown in McCoy's 5A media supplemented with 15% FBS, 2 mM L-glutamine, 100 IU/ml penicillin G, and 100 μg/ml streptomycin. The normal human fibroblast cell line GM05659 was grown in Minimum Essentials Media (MEM) with 10% FBS and 2 mM L-glutamine, 100 IU/ml penicillin G, and 100 μg/ml streptomycin. All media reagents were obtained from Invitrogen (Carlsbad, CA). The cell lines were routinely maintained at 37°C in a humidified 5% CO_2 _atmosphere.

### Reagents

The validated kinase siRNA library version 1.0 was obtained from Qiagen (Valencia, CA). Short interfering RNAs targeting TNK2, STK10, PLK1 and non-silencing control were also obtained from Qiagen (Valencia, CA). The cationic lipid transfection reagent Lipofectamine RNAiMAX was obtained from Invitrogen.

### High Throughput RNAi Screening

High-Throughput RNAi (HT-RNAi) was performed using the validated kinase siRNA library version 1. This library includes siRNAs to 572 kinases with two siRNAs per gene. Stock siRNA was diluted in siRNA buffer (Qiagen) and 9.3 ng of siRNA was printed onto white Corning 384-well plates (Fisher Scientific; Pittsburgh, PA). HT-RNAi was done by reverse transfection of cells as described previously [17]. Briefly, diluted Lipofectamine RNAiMAX reagent (Invitrogen) in OptiMEM (Invitrogen) was added to the wells and allowed to complex with siRNA for 30 min at room temperature. Ewing's sarcoma cells were resuspended in growth media without antibiotics at a final concentration of 750 cells/well for TC-32 and TC-71 or 1000 cells/well for SK-ES-1, RD-ES and GM05659. Plates were incubated at 37°C with 5% CO_2_. After 96 hours total cell number was determined by the addition of Cell Titer Glo (Promega, Madison, WI) and relative luminescence units (RLU) were measured using an EnVision plate reader (Perkin-Elmer, Wellesley, MA). Raw RLU data was used to calculate viability relative to control wells.

### Screening Data Analysis

The screening data was normalized using the standard Z-score method by correcting the raw data for plate row variation, and then normalizing and pooling data from all assay plates. The assumption is that the majority of the siRNAs are non-hits and the null distribution is normal [20]. The criteria for identification of potential hits used a Z-score cutoff of less than -1.65, which corresponded to a p-value of 0.05, in both screens for each cell line.

### Quantitative real-time PCR

Cells were transfected with 16 nM of TNK2 and STK10 siRNA or non-silencing siRNAs in 6 well plates by reverse transfection as described above. Cells were treated with siRNA for 48 hours and RNA was extracted using standard procedures. qRT-PCR using Taqman probes (Applied Biosystems, Carlsbad, CA, USA) was performed as described previously [17]. For all experiments, GAPDH gene was used as an internal control. The relative quantification was given by the Ct values, determined for triplicate reactions for test and reference samples for each target and for the internal control gene (GAPDH). Relative expression level was determined as 2^-ΔΔCt^, where ΔΔCt = ΔCt (target sample) - ΔCt (reference sample).

### Label-free Impedance Measurement of Cell Growth

The principle of impedance measurement for monitoring cellular proliferation has been previously described by Solly *et al. *[21]. Briefly, siRNA (against TNK2 and STK10) was introduced into TC-71 cells by reverse transfection of 4,000 cells/well using RNAiMAX in triplicate wells of an ACEA 96X E-Plate (ACEA Biosciences; San Diego, CA). The attachment, spreading and proliferation of cells were continually monitored every hour up to 150 hours, and changes in impedance were acquired with the real time cell electronic sensing (RT-CES) system (ACEA Biosciences). Cell growth was determined by plotting cell index measurements versus time.

### *In Vitro *High Content Apoptotic Assay

To assess apoptosis within the cell population, TC-71 cells were seeded into 384-well plates and were treated with siRNAs for the specified time and conditions described above. Cells were incubated with 10 μl of a prepared solution containing 1X annexin V binding buffer (BD Biosciences), annexin V-FITC (BD Biosciences), Ethidium homodimer (EthD; Invitrogen), and Hoechst 33258 (Sigma Aldrich) for 20 minutes at 37°C. Images were captured using the IN Cell Analyzer 3000 (GE Healthcare) and apoptotic and dead cells were detected using the IN Cell Developer Toolbox software. Nuclear staining (Hoechst) was used to identify and quantify total cell number. An image field was captured from each replicated well and cells from three wells were totaled and analyzed. Total number of cells labeled with annexin V was compared to the total number of cells as determined by Hoechst staining and the data was expressed as a percentage of Annexin V stained cells.

## Results

### RNAi screening for the identification of vulnerable "Achilles Heel" targets in Ewing's sarcoma cell lines

**Figure 1:**
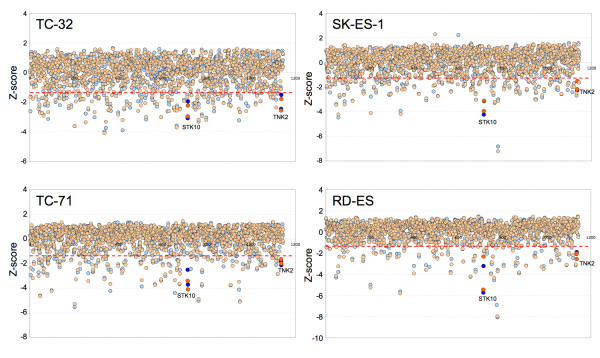
**RNAi screening of Ewing's sarcoma cell lines**. Ewing's sarcoma cell lines TC-32, TC-71, SK-ES-1 and RD-ES were transfected with siRNA targeting 572 kinases and assayed for cell viability at 96 hr as described in Material and Methods. Assays were done in duplicate and Z-score values for each siRNA treatment was plotted. Each circle represents data for one siRNA and the two colors represent data from the duplicate runs for the same cell lines. The dotted red line indicates the cut off for the significant Z score (-1.65 SD). The darkened circles correspond to the data for chosen hits STK10 and TNK2.

In order to identify genes that modulate the growth and survival properties of Ewing' sarcoma cells, we performed loss-of-function screening using high throughput RNAi (HT-RNAi) on four Ewing's sarcoma cell lines. We chose two Type I Ewing's sarcoma cell lines (TC-32 and TC-71) and two Type II Ewing's sarcoma cell lines (RD-ES and SK-ES-1) for the HT-RNAi screening. A robust HT-RNAi assay was developed and optimized that allowed for high efficiency siRNA transfection of all four Ewing's sarcoma cell lines by cationic lipids in 384-well plates (see Additional file 1: Figure S1). The HT-RNAi screen involved transfecting the Ewing's sarcoma cells with siRNA from a validated siRNA library targeting 572 kinases. Ninety-six hours post transfection, cell viability was assessed using a luminescence-based cell viability assay and the data was normalized and analyzed using Z-score method as described in Materials and Methods [20]. Duplicate runs of the HT-RNAi screens were conducted for each cell line and results are shown as dot plots of the Z-score values (Figure 1). Significant siRNA hits were classified as being 1.65 S.D. from the median. Z-score values for all individual siRNAs for the kinase screens are listed in the Additional file 2.

**Figure 2:**
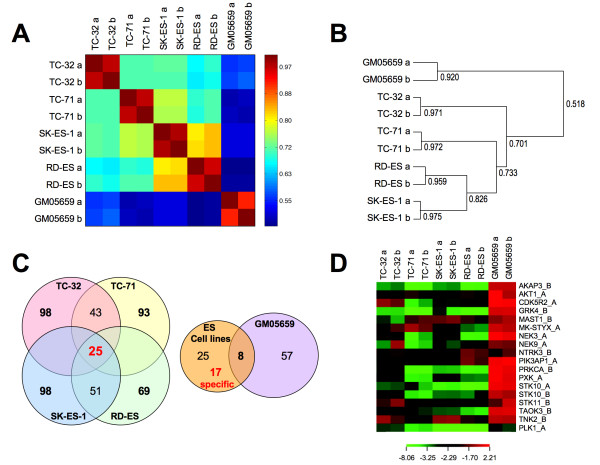
**Phenotypic profiling of kinases**. (A) Heat map showing the correlation between each independent RNAi screen for the four Ewing's sarcoma cell lines and the normal fibroblast cell line GM05659; Z-scores were used for the generation of the Heat Map. (B) Dendogram showing the correlation coefficients between all the independent siRNA screening runs; Z-scores were used for calculating the correlation coefficients. (C) Venn Diagram on the left depicts the number of significant siRNA hits for each cell line chosen based on significant Z-scores from both the independent runs for every Ewing's sarcoma cell line. Venn diagram on the right shows the overlap of significant siRNA hits between the normal fibroblast cell line and genes significant across all four Ewing's sarcoma cell lines (D) Heat map of Z-score values for the 17 siRNA hits and a PLK1 siRNA. This Heat map was generated using CIMminer http://discover.nci.nih.gov/.

Comparison of the Z-score values for each individual cell line screen shows very good correlation between the duplicate screens. Similar HT-RNAi screens were performed using normal human fibroblast cell line, GM05659 (data not shown), for comparison to Ewing's sarcoma cell line data. A significant similarity between the four Ewing's sarcoma cell lines was observed when compared to the normal fibroblast cell line GM05659 as shown using a heat map plot (Figure 2A) and dendrogram (Figure 2B). These data show the robustness of the phenotypic profiling differentiating Ewing's sarcoma cells from fibroblasts as well as two closely related subtypes of Ewing's sarcoma cell lines. The number of significant hits for each Ewing's sarcoma cell line and overlapping hits are shown in a Venn diagram (Figure 2C) showing that silencing of 25 siRNAs were significant across all four cell lines. Comparison of the overlapping Ewing's sarcoma hits with the normal fibroblast cell line showed that 17 siRNAs (corresponding to 16 genes) are specific for the Ewing's sarcoma cells. Heat map of the Z-scores shows specificity of these 16 siRNA for decreasing cell number in Ewing's sarcoma cells only as opposed to a global lethal siRNA targeting PLK1 (polo like kinase 1) that also reduces proliferation of normal fibroblast cells (Figure 2D). Of the 16 significant gene hits that modulated the growth and proliferation of Ewing's sarcoma cell lines, two genes STK10 (serine threonine kinase 10) and, TNK2 (tyrosine kinase, non- receptor, 2) were prioritized for further confirmation since both siRNAs targeting these genes were hits across all four Ewing's sarcoma cell lines (Figure 1). Confirmation also included siRNA to PLK1 as a general lethal positive control gene for comparison.

### Confirmation of the effects of STK10, TNK2 and PLK1 silencing on growth and survival of Ewing's sarcoma cells

**Figure 3:**
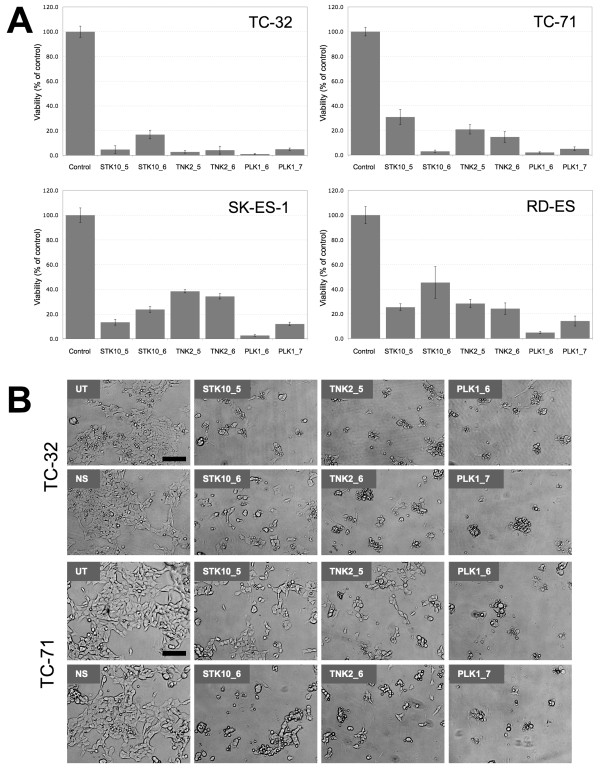
**Confirmation of the siRNA screening results**. (A) Validation of the effects of STK10, TNK2 and PLK1 silencing on cell growth of TC-32, TC-71, SK-ES1 and RD-ES cells using two siRNAs per gene. Ewing's sarcoma cells were transfected with siRNA by reverse transfection in 384-well microtiter plates and grown for 96 hrs. Each siRNA treatment was performed in quadruplicate wells. Cell number was assessed as described in Material and Methods. Treatment of Ewing's sarcoma cells with siRNA targeting STK10, TNK2 and PLK1 significantly decreased cell viability (p < 0.0001) compared to non-silencing siRNA controls. Data is representative of three independent experiments. (B) Representative images of TC-32 and TC-71 Ewing's sarcoma cells treated with siRNA shows decreased cell number and increased cellular toxicity. Bar represents 100 μm.

**Figure 4:**
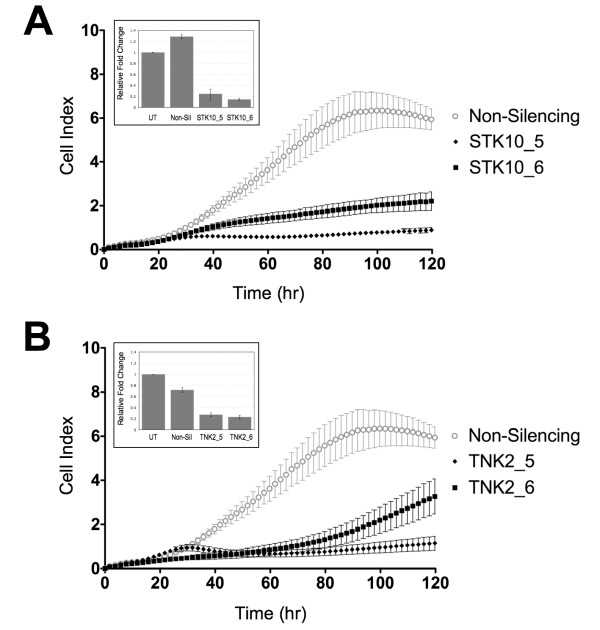
**STK10 and TNK2 knockdown leads to decreased cell viability of Ewing's sarcoma cells**. TC-71 cells were transfected with siRNAs against STK10, TNK2 or non-silencing control. Growth was assessed by impedance measurements at 1-hour intervals and cell index was plotted as a function of time. (A) Treatment of TC-71 cells with non-silencing siRNA or two different TNK2 siRNA showed reduced cell growth in cells with TNK2 knockdown (B) Treatment of TC-71 cells with non-silencing siRNA or two different STK10 siRNA showed reduced cell growth in cells with STK10 knockdown. Data is representative of three independent experiments. Confirmation of gene silencing was shown by qRT-PCR for (A inset) STK10 and (B inset) TNK2. Each individual siRNA was reverse transfected into TC-71 cells and incubated at 37°C for 48 hours. RNA was extracted 48 hours later and cDNA was prepared using random primers. qRT-PCR was done using specific TaqMan probes and all the results were normalized to GAPDH expression.

We confirmed the effects of silencing of STK10, TNK2, and PLK1 on growth and survival of Ewing's sarcoma cells by repeating the cell based assay in 384-well plates using a different lot of siRNA having the same sequences as the kinase library siRNA. Silencing of STK10, TNK2 and PLK1 by both siRNA sequences inhibited cell growth in the four Ewing's sarcoma cell lines as measured by cell number (Figure 3A). siRNA against STK10 and TNK2 showed significant reduction in cell growth in both TC-32 and TC-71 cells. The effect of STK10 and TNK2 knockdown on cell growth was very similar to the effect of PLK1 knockdown in these cells (Figure 3B). We next conducted the real-time kinetic analysis to determine the effect of STK10 and TNK2 siRNA treatment on Ewing's cancer cells using label-free impedance growth assays (Figure 4). The impedance analysis showed that the treatment of TC-71 cells with STK10 and TNK2 siRNA resulted in a very potent and sustained decrease in cell number compared to non-silencing siRNA treatment (Figure 4). To demonstrate the silencing efficiency of the siRNAs targeting TNK2 and STK10, TC-71 cells were transfected with either the specific siRNAs or non-silencing siRNA and incubated at 37°C for 48 hours. qRT-PCR was performed using Taqman probes for both genes and GAPDH was used as an internal control in both the experiments. Fold change was calculated using the Delta Delta Ct method and the results showed that at least 70% knockdown was observed using specific siRNAs against STK10 and TNK2 (Insets in Figure 4). Furthermore, treatment of TC-32 cells with siRNA targeting STK10 and TNK2 showed decrease in proteins levels compared to untreated cells or non-silencing siRNA treated cells (see Additional file 1: Figure S2). These results confirm that the siRNAs targeting STK10 and TNK2 led to specific gene knockdown in our experiments.

### Gene Silencing of STK10 and TNK2 induces apoptosis

**Figure 5:**
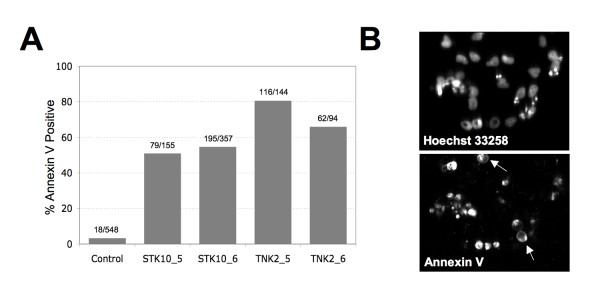
**STK10 and TNK2 knockdown induces apoptosis in Ewing's sarcoma cells**. TC-71 cells were transfected with siRNAs against STK10, TNK2 or non-silencing control. (A) STK10 and TNK2 knockdown by siRNA induces apoptosis in Ewing's sarcoma cell lines. Bar graph showing the increase in annexin V staining in Ewing's sarcoma cells treated with siRNA targeting STK10 and TNK2 compared to non-silencing control siRNA. Number of cells labeled with annexin V over total number of cells as determined by Hoechst staining from three image fields is shown above the bar for each siRNA treatment. (B) Representative image showing Hoechst stained nuclei of TC-71 cells treated 72 hours with STK_6 siRNA and corresponding Annexin V stained image.

We next examined the effect of STK10 and TNK2 siRNA treatment on the induction of apoptosis in TC-71 cells using a high content, image based analysis of annexin V staining. Image based analysis showed that treatment of TC-71 cells with siRNA targeting TNK2 and STK10 increase annexin V staining compared to cells treated with negative control non-silencing siRNA (Figure 5). These data indicate that the knockdown of STK10 and TNK2 induce apoptosis of Ewing's sarcoma cells. Representative images from the cells treated with TNK2_6 siRNA show various apoptotic bodies associated with TNK2 silencing (Figure 5B).

## Discussion

Ewing's sarcoma is a disease that appears to be etiologically driven by a few primary genetic abnormalities involving a fusion of an EWS family member with a transcription factor, of which the commonly fused transcription factor partner is *FLI1*. Therefore, these tumors offer a relatively homogenous model system for the identification of specific contextual vulnerabilities that could be targeted with novel therapeutic strategies. An improved understanding of the molecular biology of Ewing's sarcoma and the underlying genetic context has led to clinical trials of several novel therapies specifically designed to thwart critical pathways responsible for this malignancy [22]. Understanding how and when to integrate such therapies into clinical practice, although challenging, may lead to a paradigm shift towards more personalized therapy.

In recent years, there have been various independent studies looking at several different kinases and their role in sarcoma cell survival as well as their potential to be developed into specific therapeutics. In a study by Andersson *et al. *it was shown that proliferation of Ewing sarcoma cell lines is suppressed by the receptor tyrosine kinase inhibitors gefitinib and vandetanib [23]. Similarly, anti-tumor activity of GSK1904529A, a small-molecule inhibitor of the insulin-like growth factor-I receptor tyrosine kinase was reported in Ewing's sarcoma [24]. In some other studies, kinases such as JNK, TOPK, AURKA, AURKB and LYN have all been studied in Ewing's sarcoma [25-27].

We undertook this study with the goal of identifying kinases that can be targeted to modulate Ewing's sarcoma cell growth and survival. By conducting phenotype profiling of human kinases using HT-RNAi screening, we were able to obtain a better global understanding of contextual vulnerabilities in Ewing's sarcoma. We developed robust siRNA-screening assays for four Ewing's sarcoma cell lines, TC-32, TC-71, SK-ES-1 and RD-ES and performed HT-RNAi screens to generate data on the growth inhibiting effect of targeting 572 kinases (Figure 1). These data were compared to a data set from the normal fibroblast cell line GM05659 and showed stronger correlation between the Ewing's cell lines versus the normal fibroblast cells. This observation demonstrated that the two different types of Ewing's sarcoma cell lines could be grouped based on phenotypic profiling. Gene lists were compiled to identify growth-inhibiting targets in Ewing's sarcoma cells (see Additional file 2 and Table 1). We identified 25 siRNAs that were "hits" across all four Ewing's sarcoma cell lines and 17 of these siRNAs were unique to the Ewing's sarcoma cell lines when compared with the normal fibroblast cell line data (Figure 2C and 2D). These 17 siRNAs represent 16 genes since both the siRNAs targeting STK10 were on the list. Several of these genes "hits" have already been reported to have association with Ewing's sarcoma. For example, AKT1, is a downstream kinase of phosphoinositide 3-OH kinase and has been shown to prevent apoptosis and support survival of many cell types including Ewing's sarcoma [28]. Another target gene, MK-STYX is expressed in ESFT samples and was shown to be a target of EWS-FLI1 by chromatin immunoprecipitation. MK-STYX encodes for a phosphatase-dead dual specificity phosphatase-like protein implicated in the regulation of MAP kinases [29]. The actual function of STYX proteins is not known but it is suggested that they bind to phosphorylated kinases, thereby preventing de-phosphorylation by active phosphatases keeping the kinases in an active state [30]. Our results show that MK-STYX knockdown reduces cell survival in Ewing's sarcoma cells. One other target NTRK3 is the transcription factor component of common translocation fusion protein, ETV6-NTRK3, which occurs commonly in congenital (infantile) fibrosarcoma and cellular mesoblastic nephroma [31].

**Table 1:** Gene targets identified by RNAi screening as important in growth of Ewing's sarcoma cells.

Gene Symbol	Entrez Gene #	Gene Name
AKAP3	10566	A kinase (PRKA) anchor protein 3
AKT1	207	v-akt murine thymoma viral oncogene homolog 1
CDK5R2	8941	cyclin-dependent kinase 5, regulatory subunit 2
GRK4	2868	G protein-coupled receptor kinase 4
MAST1	22983	microtubule associated serine/threonine kinase 1
MK-STYX	51657	STYXL1 serine/threonine/tyrosine interacting-like 1
NEK3	4752	NIMA (never in mitosis gene a)-related kinase 3
NEK9	91754	NIMA (never in mitosis gene a)- related kinase 9
NTRK3	4916	neurotrophic tyrosine kinase, receptor, type 3
PIK3AP1	118788	phosphoinositide-3-kinase adaptor protein 1
PRKCA	5578	protein kinase C, alpha
PXK	54899	PX domain containing serine/threonine kinase
STK10*	6793	serine/threonine kinase 10
STK11	6794	serine/threonine kinase 11
TAOK3	51347	TAO kinase 3
TNK2	10188	tyrosine kinase, non-receptor, 2

*Both the siRNAs for this gene showed significant growth inhibition.

Two kinase inhibitors in clinical trails for several different cancer types are gefitinib (targets EGFR) and vandetinib (antagonist of VEGFR, EGFR and RET-tyrosine kinase). In our screens, siRNAs to EGFR and RET-kinases did not lead to significant reduction in proliferation and our siRNA library unfortunately did not include VEGFR siRNAs. Additionally, IGF1 and IGF1R were not on our siRNA library but we tested siRNAs for IGF1R, which showed inhibition of cell growth in all the four cell lines (see Additional file 1: Figure S3). Interestingly, siRNAs against AURKB led to significant reduction in growth of type II cell lines (SK-ES-1 and RD-ES) while the type I cell lines (TC-32, TC-71 and the normal fibroblast cells lines remained unaffected. The siRNA against LYN, TOPK and JNK did not affect Ewing's sarcoma cell proliferation while siRNA against AURKA had only a marginal effect.

The list of hits from our siRNA phenotypic profiling of Ewing's sarcoma cells indicates potential targets that could be exploited clinically. Over the last years, several inhibitors against targets in Table 1 have been developed. Inhibitors against AKT (i.e. MK2206 [32], GSK690693 [33]) are in early phase clinical trials. An inhibitor against PRKCA and other PKC isoforms, PKC412, has been tested extensively in the clinic already [34] and this could be a promising lead. Other PKC targeting drugs are available as well, mostly for experimental purposes [35]. Additional targets may be worthwhile exploring such as CDK5R2. There are no direct inhibitors against CDK5R2, which is a regulatory subunit of CDK5. However, we recently reported a Phase I clinical study with a well tolerated oral multi-CDK inhibitor that potently inhibits CDK5 [36]. Therefore, with an increasing number of inhibitors becoming available, hit lists from RNAi screens can directly inform translational research and drug development.

In this study, we chose three genes STK10, TNK2 and PLK1 for further validation studies as these genes were prioritized by having significant Z-score values for both siRNAs across all screens in the four Ewing's sarcoma cell lines. We confirmed that PLK1 knockdown led to increased cell death, but did not appear to be specific to Ewing's sarcoma cells as it was also a significant "hit" for normal fibroblasts (Figure 2D). Furthermore, PLK1 has been shown to be involved in cell death processes for many other different types of cancers, including rhabdomyosarcomas, osteosarcomas, hepatocellular carcinomas, esophageal carcinomas as well as hematological malignancies and in this study we intended to focus on novel targets for Ewing's sarcoma [19,37-43].

The two other promising targets identified from this RNAi screen were STK10 and TNK2. Our results clearly showed that both these genes are involved in Ewing's sarcoma cell growth and survival and are anti-apoptotic (Figures 3, 4, and 5). These results suggest that both STK10 and TNK2 would be promising kinase targets for therapeutic intervention in Ewing's sarcoma. Recently, several studies by Grueneberg and colleagues have shown that various different types of cancer cells depend on different and specific kinases for cell survival. They successfully studied "kinomes" in cervical, lung and renal cells. On browsing their target gene lists we did not see STK10 and TNK2 as "hits" in any of their screens, which also points to the fact that these two targets might be specific to Ewing's sarcoma [44-47]. Mining of gene-expression data indicate that both STK10 and TNK2 are not highly over-expressed in Ewing's sarcoma [48], hence over-expression of these genes may not be a driver for their functional specificity in this disease. STK10 belongs to the Ste20 family of serine/threonine kinases plays an important role in numerous cellular functions such as growth, apoptosis, and morphogenesis [49,50]. This protein has not been associated with cancer and most of the previous reports have studied its expression in T-cells, lymphocytes and hematopoetic tissues [51,52]. STK10 is a human homolog of murine Lok, a serine/threonine kinase highly expressed in lymphocytes. STK10 can associate with PLK1 in cells and can phosphorylate PLK1 *in vitro *[50] and engineered NIH-3T3 cell lines that over-express a dominant negative version of STK10 display an altered cell cycle phenotype characterized by increased DNA content, which raises the possibility that expression of a dominant negative STK10 may impinge upon PLK1 function *in vivo *and it has previously been shown that unregulated expression of PLK1 can result in a variety of nuclear defects. These observations are in accordance with our data, wherein we show that STK10 knockdown leads to increased apoptosis and cell death of Ewing's sarcoma cells. Our results also show that the normal fibroblast cells do not depend on STK10, as there is minimal cell death after STK10 knockdown in these cells. Although, there have been no previous reports discussing the role of STK10 in sarcomas, our results clearly demonstrate an important role for STK10 in growth and survival of Ewing's sarcoma cells.

Next, we validated the results for TNK2 knockdown and similar to STK10, TNK2 also led to increased cell death and apoptosis. TNK2, also known as ACK1 binds specifically to Cdc42 (a member of the Rho family of GTPases). Cdc42, like other Rho family members, is involved in transducing oncogenic signals from Ras to develop a transformation phenotype in mammalian cells. Pathway analysis conferred that TNK2 has been correlated with several different growth signaling pathways and is known to regulate some of the most important growth regulators in cancer cells (see Additional file 1: Figure S4). TNK2 has been shown to be involved in cell migration and induction of metastasis in transformed cells [53]. TNK2 also activates JNK and p38 mediated signaling pathways, which lead to induction of gene expression [54,55]. Recently, Howlin *et al *have shown that TNK2 preserves epidermal growth factor receptor expression on the cell surface and enhances migration and invasion of human breast cancer cells, but TNK2 did not affect apoptosis of the cells [56]. This is contrary to our observation in Ewing's sarcoma cells, wherein we showed that TNK2 knockdown is indeed responsible for causing cell death through apoptosis. These differences in TNK2 function might be attributed to the different cell types under investigation. Nonetheless, it is interesting to note that all the functions attributed to TNK2 so far point to the fact that this gene might play a significant role in the development and progression of cancer.

## Conclusions

In conclusion, this is the first study demonstrating the use of phenotypic profiling and high throughput RNAi screening to identify novel kinase targets for Ewing's sarcoma. Using this powerful approach, we were able to identify and validate two kinases, STK10 and TNK2, which have the potential to be targets for disease specific therapeutics.

## Abbreviations

FLI1: Friend Leukemia Integration Site 1; STK10: Serine threonine kinase 10; TNK2: tyrosine kinase non- receptor 2; PLK1: Polo like kinase 1; qRT-PCR: Quantitative real time polymerase chain reaction; GAPDH: Glyceraldehyde phosphate dehydrogenase; RLU: Relative Luminescence Units; RNAi: RNA interference; HT-RNAi: High throughput-RNA interference; siRNA: short interfering RNA

## Authors' contributions

DOA and SM were responsible for the initial conception and design of this study. DOA and SA were responsible for planning of the experiments. IMG performed RNAi screening and data was analyzed by CS and AC. Functional validation of siRNA data; RTH, IMG and SA performed confirmation screening and the validation of gene silencing experiments. RT provided clinical input of the experiments. DOA, SA and RT were involved in the writing of the manuscript. All authors have read and approved the final manuscript.

## Competing interests

The authors declare that they have no competing interests.

## Supplementary Material

Additional file 1 **Supplementary Figures**. File contains the following figures: **Figure S1**. Transfection optimization data showing efficacy of the selected transfection reagent and ratio on the four Ewing's sarcoma cell line; **Figure S2**. Confirmation of protein knockdown by siRNA; **Figure S3**. Effect of IGF1R silencing on Ewing's sarcoma cells; **Figure S4**. Interactions of TNK2. Click here for file

Additional file 2 **Z-score values for kinase siRNA library screen of Ewing's sarcoma cells**. Table showing the Z-score values of each siRNA in the HT-RNAi screen using the kinase library. Click here for file
