# Supplementary material for: RNAi phenotype profiling of kinases identifies potential therapeutic targets in Ewing's sarcoma
Source: Mol Cancer. 2010 Aug 18;9:218. doi: 10.1186/1476-4598-9-218 (PMC2933621; doi:10.1186/1476-4598-9-218)
Supplement: Additional file 1 — Supplementary Figures. File contains the following figures: Figure S1. Transfection optimization data showing efficacy of the selected transfection reagent and ratio on the four Ewing's sarcoma cell line; Figure S2. Confirmation of protein knockdown by siRNA; Figure S3. Effect of IGF1R silencing on Ewing's sarcoma cells; Figure S4. Interactions of TNK2. [file 1476-4598-9-218-S1.PDF]

Additional File 1: Supplementary Figures

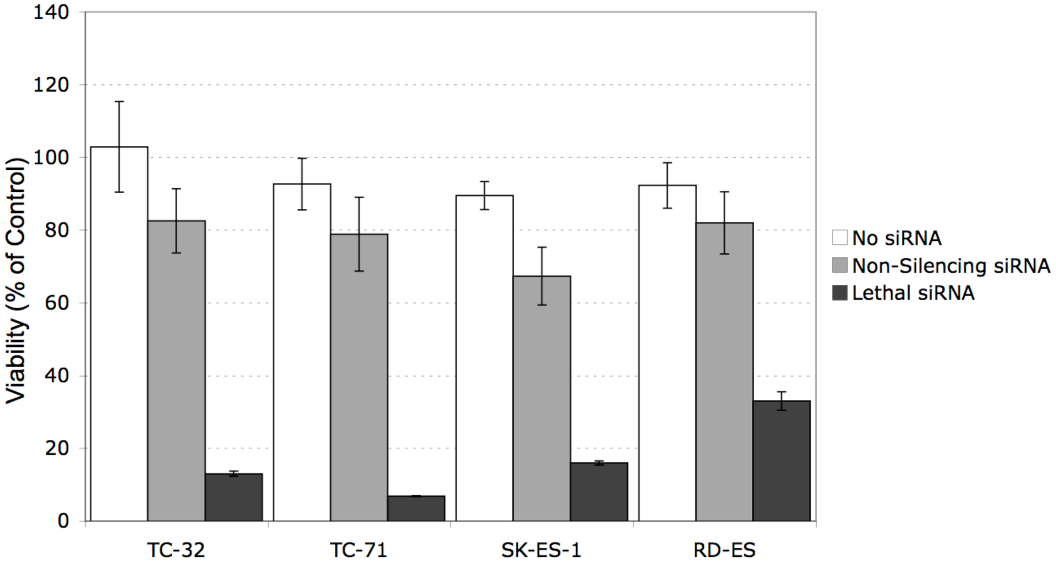

Figure S1. Optimization of siRNA transfections in Ewing's sarcoma cell lines. Transfection optimization included treatment of Ewing's sarcoma cell lines with varying concentrations of commercially available transfection reagents and three siRNA conditions, non-silencing siRNA, lethal siRNA and no siRNA. Briefly, 9.3 ng of siRNA is printed onto 384-well plates. Five dilutions of transfection reagents in OptiMEM (Invitrogen) are added to give final lipid:siRNA (vol:wt) ratios of 2:1, 4:1; 6:1, 8:1, and 10:1. Each condition is set up in quadruplicate wells. After 30 min of complexing, cells are added at a concentration of 1000 cells/well in growth media and incubated at 37˚C with 5% CO_2. Cell viability is assessed at 96 hr by the addition of 25 µl of Cell Titer Blue (Promega) and read for fluorescence intensity. Data is normalized to untreated wells (UT: no transfection reagent, no siRNA). Overall transfection efficiency was determined by subtracting toxicity due to non-silencing siRNA from toxicity due to lethal siRNA. The optimal condition for the four cell lines was Lipofectamine RNAiMAX at a 6:1 lipid:siRNA ration and this reagent and ratio was subsequently used for all screening with siRNA for each cell line.

A

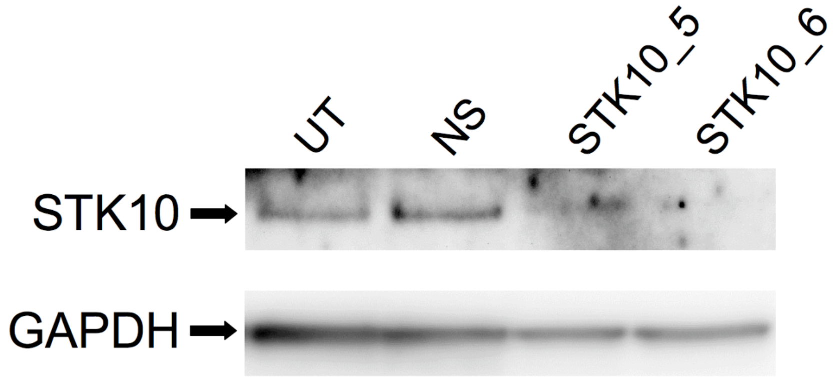

B

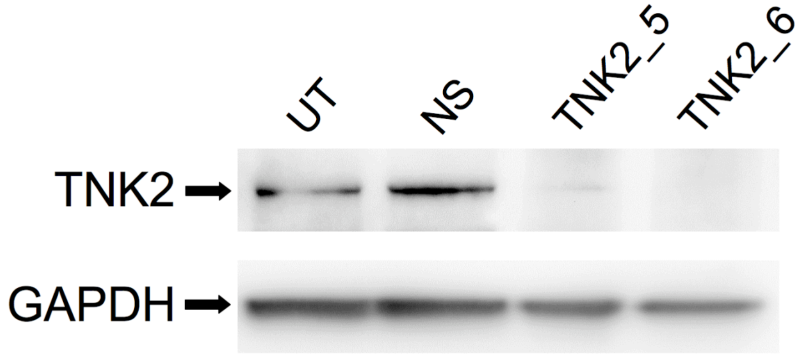

Figure S2. Confirmation of protein knockdown by siRNA. Confirmation of protein knockdown by siRNA targeting STK10 and TNK2 was shown in TC-32 Ewing's sarcoma cells. TC-32 cells were either left untreated (UT) or transfected with non-silencing siRNA (NS) or siRNA targeting (A) STK10 or (B) TNK2. Cell lysates were prepared at 48-post transfection. Equal amount of lysates were separated by SDS-PAGE and transferred to PVDF membranes for Western blot analysis. Membranes were treated with either (A) anti-SKT10 or (B) TNK2 antibodies (Santa Cruz Biotechnology, Santa Cruz, CA) and anti-GAPDH (Cell Signaling Technology) as a loading control.

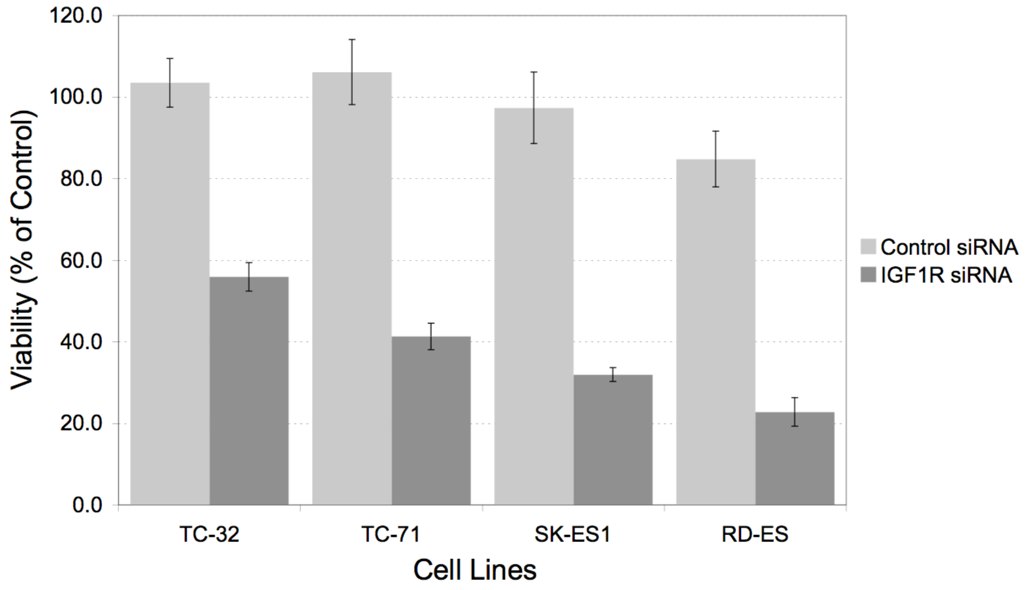

Figure S3. Effect of IGF1R silencing on Ewing's sarcoma cells. Four Ewing's sarcoma cell lines were transfected with either control non-silencing siRNA or IGF1R siRNA (Qiagen) by reverse transfection in quadruplicate wells of a 384-well microtiter plate. Cell viability was assessed at 96 hr post transfection using Cell Titer Glo (Promega). Cell viability was normalized to untreated control wells and plotted as percent of untreated controls.

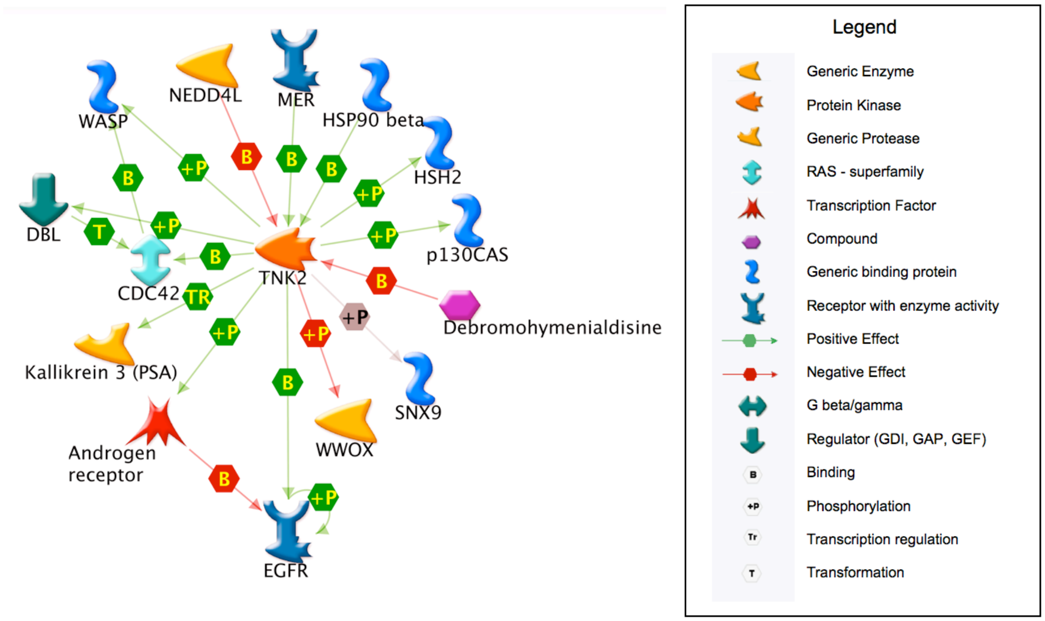

Figure S4. Interactions of TNK2. The schematic shows the proteins and compounds with known interactions to TNK2. The figure was prepared using GeneGo (Metacore Inc.).
