# Supplementary material for: RNAi phenotype profiling of kinases identifies potential therapeutic targets in Ewing's sarcoma
Source: Mol Cancer. 2010 Aug 18;9:218. doi: 10.1186/1476-4598-9-218 (PMC2933621; doi:10.1186/1476-4598-9-218)
Supplement: Additional file 2 — Z-score values for kinase siRNA library screen of Ewing's sarcoma cells. Table showing the Z-score values of each siRNA in the HT-RNAi screen using the kinase library. [file 1476-4598-9-218-S2.PDF]

Additional File 2: Z-score values for kinase siRNA library screen of Ewing's sarcoma cells

siRNA	TC-32_a	TC-32_b	TC-71_a	TC-71_b	SK-ES-1_a	SK-ES-1_b	RD-ES_a	RD-ES_b
AAK1_A	-3.57793	-3.70131	-0.86070	-1.26705	-1.78212	-2.04450	-1.13517	-1.18900
AAK1_B	-2.53570	-2.56092	-2.51738	-2.53301	-1.05795	-1.51890	-1.38335	-1.19250
AATK_A	-1.82525	-2.35760	-2.10342	-1.79362	-2.40628	-2.00224	-1.48119	-1.28424
AATK_B	-1.24760	-0.85182	-1.01418	-1.48963	-0.83995	-0.76646	-0.85811	-0.99575
ABL1_A	-0.59753	-0.53651	-1.17624	-1.16445	-0.78968	-0.72872	-0.74993	-0.73592
ABL1_B	0.42315	0.44335	0.28484	0.18298	0.55935	0.35204	0.48155	0.62453
ABL2_A	0.25927	0.50105	0.66281	0.50948	0.75476	0.73566	0.85109	1.01599
ABL2_B	0.46519	0.50809	-0.77732	-0.75461	-0.09597	-0.08360	-0.22341	-0.03929
ACVR1_A	-0.07307	0.06132	-0.02471	0.09591	-0.61256	-0.39944	0.25308	0.29027
ACVR1_B	1.08334	0.95918	0.83835	0.92801	1.09825	1.14678	0.85586	0.92557
ACVR1B_A	0.82281	0.73840	0.66913	0.65464	0.70158	0.44700	0.11817	0.07582
ACVR1B_B	-1.30689	-1.05350	-0.40044	-0.55523	-2.24403	-2.01909	-2.19268	-2.44467
ACVR2_A	-0.96294	-0.77186	0.91625	0.99439	1.13271	0.74820	0.23159	0.48037
ACVR2_B	0.16533	0.03584	-0.10817	-0.08006	-0.56519	-0.70591	-0.92374	-1.20643
ACVRL1_A	0.41181	0.18252	0.35296	0.38474	1.00036	1.03342	0.48561	0.43419
ACVRL1_B	0.39587	0.18129	0.69852	0.78071	0.28511	0.24672	0.68934	0.60497
ADCK1_A	-1.32594	-1.33663	-0.23952	-0.30631	0.12989	0.06292	-0.11662	-0.13470
ADCK1_B	-0.23158	-0.19864	0.47492	0.49530	0.61463	0.45415	0.32808	0.36922
ADCK2_A	-0.60262	0.04031	-0.83988	-0.62611	-0.16029	-0.40796	-0.69115	-1.00326
ADCK2_B	0.20009	0.14276	-0.33916	-0.67587	-0.35249	-0.36904	-0.44391	-0.28206
ADCK4_A	0.89595	0.57753	0.34255	0.28980	0.45213	0.23727	0.61207	0.62003
ADCK4_B	0.86921	0.65333	0.47602	0.52550	1.14348	0.92331	1.12789	0.97273
ADCK5_A	-1.28659	-1.02699	-2.78127	-2.64591	-2.78863	-2.65063	-2.32457	-2.33917
ADCK5_B	-2.87021	-2.91298	-2.37477	-2.40546	-1.34411	-1.44501	-0.50052	-0.70418
ADK_A	0.82316	0.88541	1.21010	0.89923	0.68045	0.59228	0.69769	0.83160
ADK_B	0.91739	0.93831	1.34116	1.11017	1.24396	1.26028	0.80654	0.96648
ADRBK1_A	0.03650	0.16851	0.30584	0.24522	0.94052	1.00602	1.21088	1.30936
ADRBK1_B	-2.61052	-1.65670	-0.12690	-0.17340	-0.17857	-0.09071	0.22994	0.34067
ADRBK2_A	-0.14686	0.05396	-0.83027	-0.71863	-1.78676	-1.52249	-0.90951	-0.68082
ADRBK2_B	0.41014	0.37963	-0.78243	-0.44772	-0.11772	-0.05939	0.27159	0.27607
AK1_A	-1.02547	-0.94101	0.17593	0.17262	-1.84614	-1.81259	-1.93640	-2.29435
AK1_B	1.17208	1.49391	0.75523	0.73701	0.86635	0.91952	0.76641	0.49709
AK2_A	-0.73642	-0.62517	0.28866	0.24782	0.84612	0.94450	0.73711	0.65307
AK2_B	0.50085	0.58142	1.00167	1.03146	1.12276	1.39133	0.89512	0.88158
AK3_A	0.81715	0.79865	0.27314	0.30520	0.03047	0.28222	0.72022	0.70562
AK3_B	-0.40236	-0.57439	0.06601	-0.13750	0.96773	1.06605	0.33198	0.41695
AK3L1_A	-2.12468	-1.89613	-0.11386	0.12679	-1.84289	-1.93195	-0.07545	-0.22172
AK3L1_B	0.60934	0.07792	-0.03680	0.23236	-0.64582	-0.45165	0.38334	0.43316
AK5_A	0.87141	1.12321	0.96696	0.89277	0.99986	0.79036	0.77900	0.57867
AK5_B	1.00341	1.20739	1.09439	1.02432	1.31243	1.32306	1.24784	0.99536
AK7_A	0.47404	0.57103	0.52257	0.12891	0.23519	0.33827	-0.18272	-0.23846
AK7_B	1.10314	1.31405	1.33651	0.95842	1.26475	1.31827	1.06268	1.12457
AKAP1_A	0.40806	0.64521	0.12250	-0.00662	-0.11226	-0.10898	-0.01133	-0.16872
AKAP1_B	0.72674	0.77212	1.20769	1.10363	-0.04282	-0.14896	0.20400	0.21674
AKAP11_A	0.06216	-0.72498	0.29356	-0.04815	0.59934	0.90367	0.88530	0.77071
AKAP11_B	0.27704	0.45968	0.02056	0.03419	0.42685	0.10908	0.31439	0.28099
AKAP12_A	-0.82416	-1.28195	-0.79054	-0.71404	-1.11221	-1.20409	0.11251	-0.30593
AKAP12_B	0.10715	0.04485	-0.40635	-0.57781	-0.11920	-0.09851	0.52325	0.40034
AKAP13_A	0.72339	0.93091	0.84484	0.87136	1.10170	1.10187	0.93903	0.81644
AKAP13_B	1.21464	1.47827	1.09789	1.13237	1.23857	1.31360	1.22471	0.92010
AKAP28_A	0.08385	0.13803	0.94271	1.13037	0.46545	0.41004	0.44189	0.50422
AKAP28_B	0.90295	0.90338	0.74680	0.77537	0.78377	0.59771	0.99538	0.88191
AKAP3_A	-1.21487	-1.34523	0.47024	0.61590	1.11764	1.07084	0.55068	0.66705
AKAP3_B	-3.31230	-3.17921	-5.01574	-4.87012	-3.30082	-3.20863	-4.55575	-4.35065
AKAP4_A	1.23791	1.21390	0.89528	0.79836	1.28357	1.10495	1.08833	1.03053
AKAP4_B	0.79930	0.52034	0.84625	0.52660	2.23363	0.25921	0.49443	0.44247
AKAP5_A	1.55426	1.39471	1.06987	1.06428	1.15205	1.19452	0.69756	0.63381
AKAP5_B	-0.55264	-1.02449	0.80678	0.57696	0.23051	0.46695	0.21961	0.26025
AKAP6_A	0.35876	0.30559	0.54384	0.57375	0.34996	0.44247	-0.27362	-0.28750
AKAP6_B	1.19776	1.31995	0.59213	0.51195	1.04085	1.01164	-0.06212	-0.09252
AKAP7_A	-2.68580	-2.83149	-2.06197	-2.18471	-1.92003	-2.02152	-3.19985	-3.90942
AKAP7_B	0.28029	0.37945	0.27452	0.03304	0.11429	0.15634	-0.07420	-0.04245
AKAP8_A	-1.46411	-1.82967	0.09633	-0.09716	0.72550	0.64762	0.20673	0.27473
AKAP8_B	-0.15431	-0.11942	0.16498	-0.00376	-0.38560	-0.14489	-0.21199	-0.22995
AKAP8L_A	0.02622	-0.00058	-0.40776	-0.63351	-0.55943	-0.48666	-0.33840	-0.21468
AKAP8L_B	-0.07098	0.19477	-0.29561	0.21764	-0.33107	-0.52080	0.31280	0.23075
AKAP9_A	-0.45027	-0.03920	-1.14732	-1.15373	-1.16222	-1.29421	-0.79680	-0.63688
AKAP9_B	-1.40700	-0.97564	-0.19277	-0.37400	-0.00519	-0.34747	-1.18947	-1.13092
AKIP_A	1.10115	0.93048	0.18107	0.29991	0.59805	0.56236	0.54467	0.63483
AKIP_B	-1.46984	-1.43513	-0.00869	0.18926	-0.32035	-0.64646	-1.58539	-1.76595
AKT1_A	-2.23141	-2.06020	-1.86569	-2.02195	-2.62925	-2.64741	-1.91067	-2.34312
AKT1_B	-3.12631	-2.67664	-0.60939	-0.76776	-1.32314	-1.10317	-0.25995	-0.48110
AKT2_A	-1.17903	-0.78231	-1.21595	-1.03828	-1.15715	-0.93891	-1.07103	-1.07629
AKT2_B	0.51947	0.86689	0.60178	0.48937	0.61383	0.87875	0.36494	0.32023
AKT3_A	0.37663	0.32448	-0.61323	-0.59114	0.21192	0.36898	0.30873	0.20764
AKT3_B	0.58159	0.78221	0.21768	0.32130	0.48739	0.64744	0.57718	0.55560
ALS2CR2_A	-1.75992	-1.62033	-0.94003	-1.06988	-2.63195	-2.48851	-0.98205	-0.92555
ALS2CR2_B	0.58588	0.44502	-0.09221	0.11196	-1.90058	-1.79058	-0.52220	-0.45520
ALS2CR7_A	-1.09051	-1.01879	-1.59037	-1.65848	-1.21590	-1.23643	-1.24720	-1.19914
ALS2CR7_B	-0.26418	-0.23155	0.07802	0.10901	0.49616	0.42007	0.85823	0.91691
ANKK1_A	0.08463	0.32019	0.56142	0.49354	-0.94734	-0.60053	0.25722	0.03529
ANKK1_B	0.46562	0.46867	0.14364	0.21359	0.44162	0.70528	0.32554	0.05679
ANKRD3_A	0.48386	0.25389	-0.33303	-0.17491	-0.74125	-0.56759	0.22151	0.25694
ANKRD3_B	0.06211	0.12180	0.69077	0.51709	-0.30424	-0.16690	0.87917	0.65443
APEG1_A	-0.99330	-0.72596	-1.46540	-1.65483	-0.92326	-0.72869	-0.82861	-0.89942
APEG1_B	1.03828	1.24191	0.72015	0.77321	0.91599	0.92981	1.02133	0.93056
ARAF_A	0.73951	0.61816	0.50946	0.51369	0.59599	0.68283	0.01171	0.19513
ARAF_B	0.02304	0.44271	0.86352	0.85399	0.72642	0.79370	0.49373	0.56843
ARK5_A	0.68438	0.71279	0.55073	0.40080	0.44194	0.74052	0.30783	0.34901
ARK5_B	0.76188	0.66749	0.78199	0.73370	0.58354	0.83467	0.67373	0.77401
ASK_A	1.00333	0.85368	-0.56708	-0.79172	-0.12464	-0.01992	-1.03457	-0.81411
ASK_B	-0.51782	-0.88777	-0.98143	-1.27046	-0.99830	-1.24026	0.12163	0.24418
ATM_A	-2.19098	-1.74026	-1.85367	-1.80796	-1.16811	-0.80889	-0.20517	-0.19703
ATM_B	0.31465	0.34724	0.13443	0.27222	-0.83980	-0.77374	-0.31487	-0.62205
ATR_A	1.13356	1.09038	0.97830	0.97807	0.79954	0.72866	0.51197	0.60704
ATR_B	-2.40113	-2.26357	-0.52874	-0.41902	-0.23839	-0.35530	-0.22794	-0.25489
AURKB_A	-0.55574	-0.48066	0.12393	0.11483	-2.25280	-2.39721	-3.18298	-2.86956
AURKB_B	-0.74838	-0.80978	-0.02422	0.00392	-1.48262	-1.79633	-1.14094	-1.10767
AURKC_A	1.11933	1.09426	0.88615	0.92382	0.85671	0.85014	0.76857	0.74952
AURKC_B	1.27816	1.47318	1.15555	1.14468	1.15888	1.11624	1.17836	1.06668
AXL_A	0.02193	-0.06805	0.65157	0.49627	0.17213	0.38092	-0.05609	0.08751
AXL_B	0.89868	0.80579	0.60059	0.07838	1.03815	1.00453	0.76727	0.68931
BCKDK_A	-0.40266	-0.69611	0.14348	0.15109	-0.25910	0.20128	-0.46784	-0.41080
BCKDK_B	0.37566	0.07131	-0.12552	0.05638	0.15943	-0.25843	-0.73154	-0.96258

siRNA	TC-32_a	TC-32_b	TC-71_a	TC-71_b	SK-ES-1_a	SK-ES-1_b	RD-ES_a	RD-ES_b
BCR_A	0.84158	0.65491	0.48929	0.52974	0.63977	0.41767	0.33063	0.47678
BCR_B	-0.88590	-1.21825	-2.70686	-2.51125	-2.06226	-1.97257	-1.55115	-1.95029
BMP2K_A	-1.47722	-1.56570	-0.53665	-1.30938	-0.01008	-0.07186	-0.57807	-0.61357
BMP2K_B	0.43498	0.43592	0.79100	0.80733	1.01550	1.14900	0.33211	0.29502
BMPR1A_A	0.24923	0.19894	0.56890	0.52936	0.63531	0.55655	-0.06603	-0.03450
BMPR1A_B	0.28235	0.35524	0.61099	0.45015	0.07175	0.03653	0.44437	0.70674
BMPR1B_A	-2.07755	-2.59724	-2.16132	-2.23492	-2.84276	-2.67156	-1.77486	-3.26595
BMPR1B_B	0.68031	0.66087	-0.14176	0.00173	0.61994	0.57775	0.37727	0.28907
BMPR2_A	-0.65573	-0.82814	-0.31979	-0.27553	0.03857	0.03580	0.36424	0.30328
BMPR2_B	0.07185	0.01066	-0.36381	-0.36586	0.05072	0.08558	0.04285	-0.21058
BMX_A	-0.21028	-0.16704	0.05563	0.48258	0.85310	0.84336	0.01473	-0.05985
BMX_B	-0.11950	0.11574	-0.06280	0.02973	-0.63324	-0.97996	-0.24183	-0.09596
BUB1_A	-1.62875	-1.40636	-0.50678	-0.81677	-1.69565	-1.90820	-0.86095	-1.11832
BUB1_B	0.80821	0.95511	0.94369	1.01491	0.97942	0.86861	0.41246	0.33975
BUB1B_A	0.01443	0.08261	-1.64588	-1.48978	-1.16798	-1.31251	-0.39393	-0.25470
BUB1B_B	0.35612	0.23355	-0.19659	-0.12173	0.40787	0.50594	0.29353	0.48198
CALM1_A	-0.05533	-0.11442	-0.11872	-0.18176	-0.59118	-0.77316	0.44426	0.52307
CALM1_B	0.17583	0.03154	-3.02729	-2.87300	-0.89878	-0.94721	-0.31734	-0.04410
CALM2_A	1.01199	1.07577	0.70420	0.88978	0.92995	0.91944	1.03064	0.97314
CALM2_B	0.07276	-0.28571	0.06823	0.34184	0.24945	0.40827	0.71344	0.76680
CALM3_A	0.84093	0.98020	0.86117	0.95846	1.04558	0.85808	0.65970	0.62371
CALM3_B	0.58331	0.48339	0.13119	0.11551	-0.13484	-0.44239	0.57177	0.76668
CAMK1_A	0.91100	0.94993	0.66503	0.62442	1.04190	0.66008	0.34532	0.53033
CAMK1_B	0.80379	0.74751	0.05571	0.30174	0.22609	0.09033	-0.05043	0.07929
CAMK1D_A	-0.52633	-0.50895	-1.15592	-0.97108	-1.23021	-1.20962	-1.32559	-1.27170
CAMK1D_B	0.24854	0.05059	0.58120	0.51166	0.51598	0.25456	0.15491	0.26543
CAMK1G_A	0.26971	0.09211	-0.12416	-0.20660	-0.29840	-0.46952	-0.63126	-0.52351
CAMK1G_B	-0.55408	-1.66851	-0.71835	-0.51837	0.25182	-1.05329	-0.91467	-0.18285
CAMK2D_A	0.43603	0.79739	0.02848	-0.03081	0.00480	0.06267	0.53893	0.58763
CAMK2D_B	1.25568	1.08256	1.18189	1.18727	0.88339	0.95620	0.62333	0.52535
CAMK2G_A	0.96479	1.16043	0.90391	0.95399	0.88033	0.81971	0.55766	0.57810
CAMK2G_B	-0.87858	-0.84244	-0.24450	-0.20313	-0.10663	-0.16798	0.22880	0.14602
CAMK4_A	1.08053	0.92814	0.91476	0.95282	1.07643	1.01338	0.81172	0.93615
CAMK4_B	0.08852	0.09260	-0.17846	0.10774	0.48978	0.52044	0.48536	0.57650
CaMKIINalpha_A	1.17645	1.28256	0.99828	1.00810	0.50267	0.61775	1.02906	1.15596
CaMKIINalpha_B	-1.76796	-1.79466	-1.14401	-1.03508	-1.40087	-1.63318	-0.84253	-1.03032
CAMKK1_A	0.78985	0.88102	0.90072	0.77598	0.91228	0.65600	0.65627	0.70760
CAMKK1_B	-1.32620	-1.25180	-0.11716	0.01237	-1.97405	-2.09350	-1.15948	-1.20101
CAMKK2_A	-1.23670	-0.97733	0.44559	0.43308	0.49281	0.62735	0.54024	0.60024
CAMKK2_B	-0.14680	-0.59490	-0.78962	-0.80434	0.43237	0.46055	0.21111	0.33556
CARKL_A	0.99219	1.10382	1.02362	1.11122	1.22283	1.18352	1.23755	1.23516
CARKL_B	0.89930	0.88420	0.74433	0.64163	0.79443	1.01560	0.69040	0.58041
CASK_A	-0.17007	-0.35754	-0.42704	-0.36659	-0.48747	-0.33743	-0.93744	-0.99679
CASK_B	-2.21099	-2.11394	0.06049	0.05773	-0.59750	-0.38381	-0.10448	-0.24671
CDC2_A	1.10397	1.18122	0.68457	0.46882	-0.00542	0.10342	0.03552	0.11121
CDC2_B	0.92740	0.95743	0.55751	0.43003	0.56088	0.24097	0.74912	1.08017
CDC2L1_A	-1.82711	-2.46436	-2.70763	-3.00406	-0.79680	-0.73703	-0.57090	-0.74933
CDC2L1_B	-0.02277	-0.11452	-1.66395	-1.94609	0.26804	0.36282	0.19431	0.36355
CDC2L5_A	0.65606	0.97776	0.38268	0.18072	0.43901	0.58897	0.47695	0.67051
CDC2L5_B	1.02241	1.11306	1.08164	0.99763	0.76481	0.87199	0.71515	0.78646
CDC42BPA_A	-1.32175	-0.57815	-0.14826	-0.97418	-2.28020	-2.38903	-2.74269	-2.58485
CDC42BPA_B	-0.51945	-0.84698	-0.68125	-0.66816	-2.48504	-2.52828	-2.27284	-2.35281
CDC42BPB_A	0.80615	0.77860	1.05932	1.07937	0.61012	0.59491	0.80172	0.66080
CDC42BPB_B	-0.10285	-0.23195	0.11569	0.13904	-0.59907	-0.63015	0.17805	0.03509
CDK11_A	0.25487	0.32236	0.28975	-0.26824	-0.63608	-0.59113	0.36589	0.41755
CDK11_B	0.24463	0.14404	1.07985	0.88197	0.68793	0.77163	0.20031	0.25676
CDK2_A	-0.94261	-0.89041	-2.84368	-2.97748	-1.28631	-1.30192	-0.79971	-1.06773
CDK2_B	1.45537	1.43814	0.95302	0.90991	1.36731	1.36768	1.07816	0.89733
CDK3_A	-0.51559	-0.61349	0.21446	0.37102	-1.06253	-0.98618	-0.38568	-0.55817
CDK3_B	-0.54000	-0.57295	-1.63797	-1.00962	-3.09938	-3.12147	-2.51496	-2.65195
CDK4_A	0.10276	0.18226	-0.99111	-0.73960	-0.74250	-0.76931	-0.50989	-0.73814
CDK4_B	-0.17754	-0.15441	-1.02606	-0.91235	-1.86638	-1.67762	-0.99877	-1.12102
CDK5_A	1.29446	1.30497	0.96160	1.00270	1.26114	1.21231	1.15152	1.03111
CDK5_B	-0.93623	-0.84266	-1.05495	-0.94975	-0.14106	-0.11777	-0.27304	-0.38184
CDK5R1_A	1.15700	1.24138	0.86869	1.02041	1.12143	1.23425	0.62983	0.78242
CDK5R1_B	-2.47914	-2.39763	0.11132	0.24680	0.34068	0.20926	-0.29795	-0.25941
CDK5R2_A	-1.51639	-1.66627	-3.85980	-3.34779	-2.71061	-2.72544	-2.47479	-2.51934
CDK5R2_B	-0.39176	-0.40216	-0.81401	-0.91851	-0.51481	-0.20165	-0.67264	-0.65128
CDK6_A	-0.80108	-0.89100	-1.28984	-1.46248	-0.90954	-0.89282	-1.20524	-1.04003
CDK6_B	0.42289	0.32307	0.96667	0.80399	0.75483	0.69133	0.61331	0.73858
CDK7_A	0.56234	0.47417	-0.02986	-0.01255	0.83629	0.84632	0.59040	0.71384
CDK7_B	-1.16119	-1.93765	-1.56411	-2.06707	-1.59942	-1.61102	-2.26432	-2.48724
CDK9_A	-1.10368	-1.25666	-0.23232	-0.32922	-1.44786	-1.37865	-0.86597	-0.58187
CDK9_B	1.34693	1.40752	0.95681	0.87216	1.15844	0.91011	0.79861	1.08930
CDKL1_A	0.43157	0.31537	-0.17358	0.03469	0.11581	0.34512	0.77854	0.66934
CDKL1_B	0.37229	0.37859	0.33381	0.38181	0.57218	0.82452	0.63248	0.55777
CDKL2_A	1.07890	0.90518	0.06063	-0.11721	0.02835	0.09765	0.83455	0.90186
CDKL2_B	1.42071	1.32195	0.60466	0.43457	0.72454	0.69329	0.74819	0.85937
CDKL3_A	-0.09307	0.30734	0.62181	0.54293	1.25225	0.32064	0.58250	0.49819
CDKL3_B	0.79293	0.95922	0.46451	0.44995	0.29327	0.48961	0.43324	0.15586
CDKL5_A	-0.07013	0.38340	0.85710	0.97830	0.52784	0.51739	1.01700	0.92073
CDKL5_B	1.11144	1.08484	0.43876	0.42069	0.29609	0.39781	0.73631	0.86792
CDKN1A_A	1.30795	1.37412	0.94967	0.91636	1.20412	1.13342	0.98599	0.89016
CDKN1A_B	0.53875	0.75768	0.86056	0.80413	0.90977	0.79766	0.47645	0.43085
CDKN1B_A	0.39278	0.34700	0.66243	0.32194	0.40688	0.18165	-0.24489	-0.19069
CDKN1B_B	-0.63578	-0.72190	-0.41270	-0.45724	0.12087	0.07257	0.00928	0.03194
CDKN1C_A	0.89486	1.10164	0.73554	0.85182	0.93124	1.06266	0.62659	1.16730
CDKN1C_B	1.03540	1.20479	1.04028	0.94654	1.10211	1.18326	1.08563	1.15517
CDKN2A_A	-1.26255	-0.69866	0.62205	0.66416	1.19390	1.13570	1.09018	1.12906
CDKN2A_B	-1.03767	-1.12501	-1.35341	-1.24514	-0.14432	-0.04459	-1.20299	-1.30267
CDKN2B_A	1.13566	1.06195	1.28241	1.44993	1.30362	1.16314	1.47049	1.43429
CDKN2B_B	0.81028	0.64786	0.57024	0.73224	1.01807	0.92089	0.79801	0.78769
CDKN2C_A	0.75487	0.81210	1.13316	1.08596	0.86757	0.99479	0.58965	0.62503
CDKN2C_B	0.60190	0.68289	0.72928	0.65151	0.21911	0.30602	0.32672	0.35406
CDKN2D_A	-0.56079	-0.53816	-1.29766	-1.11194	0.20897	0.18334	-1.23356	-1.49738
CDKN2D_B	1.13561	1.00261	0.94362	0.93954	1.08334	1.02848	0.78331	0.87460
CERK_A	0.83993	1.12111	0.75537	0.75743	1.11663	0.89477	0.90882	0.80881
CERK_B	-0.40750	-0.80530	-0.50653	-0.39240	0.24647	0.15982	0.72149	0.88770
CHEK1_A	-0.68274	-0.68948	-1.28950	-1.17110	0.03608	-0.05954	-0.20023	-0.21122
CHEK1_B	0.21058	0.31206	0.25971	0.59979	0.87787	0.94461	1.18011	1.22896
CHEK2_A	0.75435	0.59760	0.25058	0.63398	0.25960	0.35346	0.61288	0.64434
CHEK2_B	-0.16938	-0.28622	0.23195	0.42207	0.37057	0.37116	-0.00280	0.10667
CHK_A	-2.36930	-2.11166	-1.17138	-1.29654	-3.07115	-2.90372	-3.13351	-3.58012
CHK_B	0.42338	0.58024	1.14706	0.95673	0.91737	0.88206	-0.77971	-0.93366

Additional File 2: Z-score values for kinase siRNA library screen of Ewing's sarcoma cells (cont)

siRNA	TC-32_a	TC-32_b	TC-71_a	TC-71_b	SK-ES-1_a	SK-ES-1_b	RD-ES_a	RD-ES_b
CHUK_A	0.58995	0.60391	1.00877	0.98957	0.44754	0.46447	0.36984	0.43933
CHUK_B	-0.28106	-0.21417	-0.37099	-0.16488	-0.68970	-0.71110	-0.16210	-0.01684
CIB3_A	-0.36447	-0.45235	-0.97874	-0.51707	-1.02264	-1.14141	0.00986	-0.31067
CIB3_B	-0.55682	-0.67837	-0.77351	-0.58775	-1.77348	-1.66951	-1.34671	-1.39389
CINP_A	0.90527	0.81266	0.66692	0.76975	0.45488	0.54405	0.23194	0.08827
CINP_B	0.19686	0.21075	0.56150	0.59173	0.63975	0.57493	-0.00055	-0.09094
CIT_A	0.59067	0.54822	0.11611	-0.11265	0.58382	0.53112	0.00849	-0.00094
CIT_B	-0.20423	-0.24548	-0.34320	-0.92070	-0.04198	-0.26329	-0.53318	-0.47626
CKB_A	-0.31373	0.10219	-1.30952	-1.09477	-1.04210	2.29442	-0.26127	-0.50153
CKB_B	-0.25954	-0.10565	-1.08076	-1.19420	-0.87463	-0.90227	-0.41907	-0.62217
CKM_A	1.13333	1.35327	0.97956	0.83720	1.02227	1.18237	0.50046	0.64924
CKM_B	-0.16698	0.02783	-0.56665	-0.79482	0.32326	0.47419	0.23547	0.36317
CKMT1_A	0.82340	0.64372	0.68787	0.69363	0.25104	0.21355	0.33456	0.35198
CKMT1_B	-2.28632	-2.12004	-1.02168	-1.06468	-1.08576	-1.05260	-1.09095	-1.13788
CKS1B_A	-1.77718	-1.45157	0.17966	0.54033	0.11832	-0.02487	0.24423	0.40455
CKS1B_B	0.79268	0.73821	0.95398	1.10999	1.03504	0.79808	1.32291	1.37721
CKS2_A	0.21622	0.00909	-0.07352	-0.07679	-0.19026	-0.45286	0.43457	0.38980
CKS2_B	1.10225	1.15103	1.04439	0.96195	0.87740	0.92147	0.94997	0.88232
CLK2_A	-0.57256	-0.33697	-0.05559	0.17038	0.82376	0.67755	-0.46397	-0.39381
CLK2_B	-0.26636	-0.35157	-0.89552	-0.84663	-2.08425	-2.27173	-2.05660	-2.37500
CLK3_A	-1.09781	-1.10677	-0.80842	-0.71014	0.05720	-0.12406	-0.25028	-0.39945
CLK3_B	-0.46925	-0.50661	-0.22582	-0.16446	0.41384	0.24843	0.46163	0.50867
CNKSR1_A	-0.20723	0.12582	-0.06043	-0.25597	0.65664	0.69562	-0.24027	-0.24705
CNKSR1_B	0.35099	0.69207	0.75251	0.81623	0.70064	0.48799	0.19545	0.19972
CRK7_A	-0.77884	-0.91025	-0.06364	-0.38106	-0.35131	-0.43497	-0.03156	0.17137
CRK7_B	0.43920	0.48464	0.09681	0.14295	0.39139	0.28629	0.15050	0.20117
CSK_A	0.12846	0.11147	0.03363	0.05173	0.63961	0.48582	-0.34075	-0.39309
CSK_B	0.39673	0.52657	0.22757	0.46007	0.33933	0.44235	-0.19869	-0.41889
CSNK1A1_A	0.09180	-0.02337	0.41443	0.73809	-0.04212	-0.14296	0.19092	0.26286
CSNK1A1_B	-1.99465	-1.82756	0.66763	0.93804	0.49253	0.50243	-0.02118	-0.12279
CSNK1A1L_A	-0.43609	-0.35531	0.36267	0.47551	0.13707	0.40808	0.61446	0.54154
CSNK1A1L_B	-0.67482	-0.84244	-1.11615	-0.88216	-0.54054	-0.58532	0.08230	-0.30974
CSNK1D_A	-0.96343	-0.64389	-0.67149	-0.41142	-0.91333	-0.94603	-1.10914	-1.08974
CSNK1D_B	-0.40109	-0.25840	0.59846	0.83333	0.07978	0.05379	-0.29390	-0.56356
CSNK1E_A	0.76325	0.67632	0.95968	1.08304	0.63650	0.64600	0.45770	0.43878
CSNK1E_B	-0.00771	-0.26141	0.47027	0.64284	0.05612	0.13252	-0.80867	-0.97276
CSNK1G1_A	0.17652	0.17893	0.47249	0.73983	0.06038	0.05553	0.31404	0.27270
CSNK1G1_B	0.88091	0.65849	1.10033	1.15780	0.89104	1.01662	0.33980	0.41934
CSNK1G2_A	0.41963	0.18388	0.20612	0.17477	-1.09428	-1.00683	-0.54984	-0.24446
CSNK1G2_B	-0.65850	-0.60594	-0.18883	-0.26313	-0.93153	-0.97489	0.00578	0.00688
CSNK1G3_A	0.78512	0.80698	0.82804	0.84624	0.50463	0.28581	0.72310	0.91203
CSNK1G3_B	1.07181	0.96192	1.14529	1.24441	0.93311	1.04428	0.95455	1.18175
CSNK2A1_A	0.26286	0.34062	0.75726	0.62408	0.72175	0.68927	0.85975	0.88473
CSNK2A1_B	1.18166	1.12018	0.94808	0.99430	1.37273	1.24901	1.14346	1.21255
CSNK2A2_A	1.05516	1.05211	0.96767	1.02501	1.12632	1.13088	0.77055	0.63815
CSNK2A2_B	0.23287	-0.02716	0.65732	0.75699	-0.11791	-0.16379	0.19744	0.06739
CSNK2B_A	-0.11367	-0.01827	-0.19209	-0.55679	-0.59071	-0.53916	-0.27962	-0.40095
CSNK2B_B	0.73724	0.62687	0.24652	0.26685	-0.61451	-0.29707	0.08033	0.24956
DAPK1_A	1.06501	0.85866	1.04634	0.99727	0.87714	0.82185	0.63546	0.71204
DAPK1_B	-0.63775	-1.38364	-0.05190	-0.02942	-0.84278	-0.87890	-0.66526	-0.75617
DAPK2_A	-0.51393	-1.71527	-1.83558	-1.69870	-0.00173	0.01471	-0.45798	-0.35491
DAPK2_B	-1.75618	-1.63194	-0.92191	-1.21606	-1.21264	-1.36245	-1.19739	-0.97127
DAPK3_A	-1.60142	-1.31588	-0.59460	-0.56456	-0.08227	-0.21002	0.56582	0.54499
DAPK3_B	0.76931	0.91859	-0.47075	-0.41030	0.11731	0.08410	0.48127	0.50920
DCAMKL1_A	0.10939	0.13706	0.41057	0.60705	0.43256	0.54838	0.43983	0.22476
DCAMKL1_B	1.05691	1.06955	0.87702	0.97832	0.39670	0.16611	0.45691	0.45512
DCK_A	1.02658	1.14277	0.48515	0.28016	-0.06768	0.29534	0.25869	0.19587
DCK_B	1.25801	1.52257	1.30377	1.18870	1.18563	1.14819	0.69541	0.71773
DDR1_A	0.91820	0.85423	0.15964	0.29464	0.79956	0.83749	-0.30776	-0.06747
DDR1_B	0.49952	0.70403	0.50744	0.42610	0.01673	0.18865	0.30142	0.31137
DDR2_A	-0.14247	-0.32703	-1.03068	-1.23295	-0.03373	-0.07471	-0.38134	-0.31953
DDR2_B	-0.58817	-0.77594	-1.51249	-1.38083	-0.52445	-0.41026	-0.75100	-0.85192
DGKA_A	-0.38804	-0.22921	-0.04636	-0.12138	-1.37802	-1.14016	0.11483	0.12481
DGKA_B	0.72189	1.03831	0.57469	0.21808	0.68661	0.84240	1.08149	1.11961
DGKB_A	-1.51673	-1.35975	-1.61767	-1.45063	-0.70750	-0.70332	-0.62999	-0.54193
DGKB_B	-0.34653	-0.40909	0.00793	0.29574	0.18053	0.10482	0.92854	0.93770
DGKD_A	-0.67521	-0.52228	-0.10998	-0.05011	-0.24700	-0.31770	0.31661	0.26207
DGKD_B	1.02691	1.08663	0.60381	0.25981	1.21862	1.16123	0.74629	0.76895
DGKG_A	1.65149	1.60288	1.10322	1.19155	1.28095	1.29935	1.06321	1.07404
DGKG_B	0.43675	0.25658	0.96262	1.09103	0.82649	0.71088	0.88729	0.97194
DGKQ_A	0.04488	0.23357	0.59185	0.55196	0.46074	0.13164	0.29788	0.22859
DGKQ_B	-1.13337	-0.82223	-2.46660	-2.76553	-0.01834	-0.36732	-0.31689	-0.43490
DGKZ_A	0.49155	0.46129	0.57565	0.44390	1.32084	1.34553	1.11789	1.15166
DGKZ_B	1.10091	1.31599	0.67076	0.73306	1.02818	1.18017	0.55969	0.52801
DGUOK_A	0.20683	0.34805	-0.55710	-0.38831	-0.01783	0.09902	0.85453	0.79323
DGUOK_B	0.07560	0.45296	-0.61339	-0.67339	-0.42578	-0.47593	-0.81304	-1.12111
DMPK_A	0.40417	0.37632	0.60865	0.54538	1.12321	1.04091	0.06556	0.32448
DMPK_B	1.18146	0.97645	0.93745	0.88932	0.55675	0.70905	0.31218	0.42265
DTYMK_A	0.44185	0.74893	0.77324	0.61356	0.72531	1.08053	0.99019	1.02796
DTYMK_B	0.28290	0.62395	0.03308	-0.07992	-0.31431	-0.01913	0.00290	-0.01231
DYRK1A_A	0.39887	0.37497	0.40507	0.53484	0.33934	0.40911	0.48565	0.49418
DYRK1A_B	-0.61472	-0.44090	0.70486	0.42081	0.01180	0.06409	-0.14975	-0.10890
DYRK1B_A	-0.10944	-0.17026	0.23071	0.36952	0.08828	-0.10080	0.78024	0.68012
DYRK1B_B	-0.77289	-0.96950	-0.72325	-0.32809	-0.64446	-0.69274	-2.05312	-2.29114
DYRK3_A	1.25555	1.18089	1.12183	1.04217	1.49026	1.37859	1.38682	1.34437
DYRK3_B	-1.36013	-1.41345	0.10712	0.10616	-0.26200	-0.46403	-0.79012	-0.98618
DYRK4_A	-0.20278	0.17242	0.08933	0.14036	-0.76976	-0.59508	0.66023	0.50692
DYRK4_B	0.24223	0.66960	0.43871	0.33999	-0.07585	0.12400	0.39063	0.39856
EEF2K_A	-0.84552	-0.74234	-0.34036	0.22128	-1.06891	-0.91232	-0.74151	-0.96222
EEF2K_B	0.43101	0.44741	0.02392	0.04190	0.62885	0.74188	0.17318	-0.00582
EGFR_A	-0.15977	-0.29955	-1.12388	-0.95448	-0.39840	-0.55359	-0.46555	0.46643
EGFR_B	1.07496	0.92891	0.31472	0.33547	-0.19363	0.17675	-0.25633	-0.09547
EIF2AK2_A	0.25176	0.29664	0.39911	0.45176	0.52888	0.75386	0.21201	0.16488
EIF2AK2_B	1.25337	1.44114	1.30084	1.22189	1.29017	1.25710	0.79804	0.84416
EIF2AK3_A	1.44793	1.52683	0.44015	0.79495	1.10538	0.99145	1.12262	1.05776
EIF2AK3_B	-2.11528	-2.18190	-0.33902	-0.36510	-0.83079	-0.72975	-0.54508	-0.47481
EIF2AK4_A	-0.19681	0.00747	-0.02763	-0.13205	-0.71944	-0.79732	-0.57539	-0.58120
EIF2AK4_B	0.34573	0.35596	0.60559	0.64913	-0.39583	-0.43914	0.11639	0.15130
EKI1_A	0.64057	0.41611	0.50897	0.52573	0.44188	0.59535	0.79628	0.77323
EKI1_B	1.02084	0.95194	1.10103	0.99781	0.66517	0.80489	0.78764	0.76665
EPHA1_A	0.41693	0.49329	0.68566	0.45277	0.38353	0.23983	0.31896	0.46582
EPHA1_B	0.99860	0.90093	0.31926	0.61295	1.28626	1.24618	0.97974	1.01393
EPHA2_A	0.02539	-0.11949	-1.13422	-0.98459	0.01887	-0.75810	-0.08906	0.11214
EPHA2_B	0.14101	-0.03058	-0.06028	-0.30860	0.53213	0.48553	-0.02197	-0.01332
EPHA3_A	1.17858	1.38732	1.04737	1.01859	1.12131	0.94093	0.98906	1.11150
EPHA3_B	1.06031	0.92060	0.08651	0.18649	0.93920	0.85913	0.74919	0.91378
EPHA4_A	1.49887	1.38471	1.18367	0.97043	1.35045	1.27301	1.05928	1.16148
EPHA4_B	-0.91678	-0.58975	-2.04476	-2.45634	-1.94850	-1.98916	-0.97556	-0.94946
EPHA5_A	1.43884	1.44875	1.07435	1.02547	1.35878	1.31360	1.11349	1.08100
EPHA5_B	-2.28126	-1.21116	-2.31362	-2.14188	-2.31159	-2.14994	-3.22560	-3.49696
EPHA7_A	1.24755	1.37679	1.11852	1.15066	1.32505	1.41713	1.12362	1.09598
EPHA7_B	0.94526	0.98316	0.77738	0.91793	0.89344	0.64874	0.91230	0.74030
EPHA8_A	1.38681	1.44554	1.12347	1.06819	1.32685	1.37923	1.25445	1.28458
EPHA8_B	0.68784	-0.40954	0.60768	0.49817	-0.12765	0.01798	0.36414	0.35480
EPHB1_A	1.33821	1.29212	1.10715	1.15737	1.36495	1.40308	1.28816	1.34774
EPHB1_B	-0.13623	-0.18545	-0.77748	-0.60090	-1.56961	-1.50130	-2.66530	-2.09297
EPHB2_A	0.75271	0.66582	1.14263	0.89882	1.15402	1.15839	1.32977	1.29615
EPHB2_B	0.90151	0.76311	0.67572	0.69495	1.08496	1.00256	0.80887	0.56498
EPHB3_A	-1.13086	-1.14513	-0.60915	-0.76651	-1.05638	-0.99961	-2.13480	-1.97151
EPHB3_B	0.59495	0.56678	0.12351	0.13949	0.69089	0.81378	0.23648	0.37932
EPHB4_A	1.45840	1.39996	1.16016	1.07011	1.35409	1.51318	1.24654	1.13846
EPHB4_B	0.38023	0.23007	0.53005	0.44505	0.41180	0.57349	0.47101	0.59500
EPHB6_A	-0.53037	-0.69214	-0.12806	-0.04936	-0.24565	-0.31842	-0.56386	-0.46798
EPHB6_B	-0.70259	-0.53447	-0.31915	-0.30652	-0.24246	-0.11346	-0.05282	0.10692
ERBB2_A	-0.70616	-0.90047	-0.89926	-1.05641	-0.86995	-0.89454	-0.66639	-0.62728
ERBB2_B	-0.26092	-0.33146	-0.45066	-0.40472	0.25355	0.10475	0.07894	0.15065
ERBB3_A	-0.48792	-0.45877	0.35580	0.41391	-0.63341	-0.45604	-0.18098	-0.34991
ERBB3_B	-1.33132	-1.24757	-0.87430	-0.80727	-1.79906	-1.68074	0.05025	0.03004
ERBB4_A	0.09999	0.16363	-0.39786	-0.40297	-0.76238	-0.51312	-0.23658	-0.21800
ERBB4_B	0.47018	0.52923	0.58626	0.57781	-0.25665	-0.08379	0.31250	0.33372
ERK8_A	-0.91632	-0.90418	-0.27074	-0.13810	0.88064	0.84288	0.89689	0.92506
ERK8_B	-0.30348	-0.33829	-0.95139	-1.02138	-0.21119	-0.24898	-0.41471	-0.55097
ERN1_A	1.00030	0.99183	0.47272	0.60218	1.16906	1.13703	0.65968	0.55242
ERN1_B	0.39922	0.29836	0.74102	0.87696	0.56532	0.32364	-6.88824	0.10073
FASTK_A	-0.06733	-0.21421	-0.45959	-0.55410	-0.34024	-0.31587	0.28043	0.12137
FASTK_B	0.41344	0.31187	-0.43137	-0.42044	0.11158	0.19222	0.34385	0.23691
FER_A	-0.13907	-0.20896	-0.65824	-0.89750	-0.90761	-0.76562	0.38465	0.20796
FER_B	-0.36203	-0.35869	-0.98495	-0.97862	-0.72639	-0.70130	0.11757	0.05465
FES_A	-0.74628	-1.04280	-1.36193	-1.30785	-0.94244	-0.95781	-0.26980	-0.11129
FES_B	0.50701	0.52137	-0.34540	-0.29561	0.46504	0.60507	-0.35926	-0.15167
FGFR1_A	1.08682	0.90254	0.42746	0.37648	0.43701	0.35586	0.59406	0.72451
FGFR1_B	0.47853	0.63896	0.56043	0.64204	0.81217	0.79618	0.54614	0.47382
FGFR2_A	0.96551	0.98994	0.70695	0.69083	0.91880	0.97898	0.98809	0.93078
FGFR2_B	-0.29953	-0.13703	-0.80731	-0.69776	-0.80287	-0.75280	-1.31750	-1.32786
FGFR3_A	0.98868	0.97070	0.74432	0.87509	0.73068	0.88388	0.39747	0.33376
FGFR3_B	0.61855	0.45409	0.09683	0.21750	0.68302	0.65679	0.58416	0.77666
FGFR4_A	-1.02761	-0.98091	-0.98611	-0.76700	-0.61131	-0.43808	0.04777	-0.12858
FGFR4_B	-1.48215	-1.57827	-2.47815	-2.36201	-0.98696	-0.99394	-0.84080	-0.76675
FLJ11149_A	-2.15128	-2.60133	-1.01601	-1.41343	-1.50167	-1.78843	-0.60613	-0.19272
FLJ11149_B	1.25490	1.31275	0.42518	0.50875	0.88591	0.79060	0.77542	0.88996
FLJ13052_A	1.08495	1.28804	0.93408	1.04774	1.17674	1.09936	0.91387	0.98461
FLJ13052_B	-0.64428	-0.68064	-0.30366	-0.18506	0.66098	0.68738	-0.11900	-0.22593
FLT1_A	0.08739	-0.04113	-0.03166	0.05830	0.28987	0.24556	-0.02246	-0.23124
FLT1_B	0.37269	0.27384	-1.18611	-0.91166	-1.36571	-1.35667	-0.73850	-0.86690
FLT3_A	-1.36409	-1.32858	-1.66361	-1.15424	-1.42230	-1.13758	-1.17739	-1.30781
FLT3_B	0.05981	-0.22819	0.15111	0.04436	-0.30639	-0.09831	0.07177	-0.21286
FLT3LG_A	0.57228	0.64015	0.77316	0.73617	0.81584	0.63480	0.66230	0.29714
FLT3LG_B	-0.05680	-0.02123	-1.90933	-1.81502	-0.03958	-0.12556	-0.23875	-0.27137
FLT4_A	0.07080	0.10130	-0.86151	-0.92654	-0.67060	-0.75723	-0.21441	-0.31495
FLT4_B	-0.60894	-0.60319	-0.09259	-0.15397	1.01730	0.77429	0.19233	0.28492
FN3K_A	-0.34632	0.04546	0.75773	0.47455	0.22122	0.05480	0.70497	0.44614
FN3K_B	-1.72369	-1.66320	-0.27211	-0.31781	-1.26727	-1.55714	-1.30335	-1.50429
FRK_A	-1.37023	-1.39698	0.47421	0.39711	-0.06558	0.22593	-0.32848	-0.37903
FRK_B	-0.27963	-0.26047	0.86617	0.89262	0.50216	0.31948	0.59495	0.62856
FUK_A	-0.29592	0.02914	0.55927	0.59815	0.86257	0.89093	0.95411	1.01926
FUK_B	-0.60726	-0.23595	-0.11201	0.03577	-0.40978	-0.35173	-0.64703	-0.70335
FYN_A	-0.95098	-0.73002	0.80746	0.69794	0.55837	0.68536	0.98926	1.00467
FYN_B	-1.45097	-1.47331	-1.14251	-1.06090	-0.29745	-0.08616	-0.42186	-0.03134
GAK_A	-0.53982	-0.34462	0.02986	-0.07089	-0.14441	-0.21254	-0.56811	-0.46053
GAK_B	-0.02804	0.11251	-0.72050	-0.82942	-0.56089	-0.58896	-0.14401	-0.23906
GIT1_A	0.33846	0.70051	0.93594	0.99301	0.86687	0.83599	0.38916	0.44059
GIT1_B	-0.42452	-0.22967	0.37443	0.36722	-0.30448	-0.44342	0.36427	0.54894
GIT2_A	0.11179	-0.22958	0.10247	0.13289	0.56638	0.76569	0.23503	0.21848
GIT2_B	-0.22384	-0.47196	-2.01092	-2.14753	-0.94018	-1.03071	-0.12075	0.15839
GK_A	-0.70156	-0.64586	0.18416	-0.08385	-0.93908	-0.90166	-0.26385	-0.29072
GK_B	-0.16029	-0.54788	0.46715	0.47800	0.18927	0.38435	0.39768	0.35673
GKAP42_A	-0.53901	-0.81486	-0.49392	-0.12381	-0.18788	-0.15650	-0.79983	-1.00727
GKAP42_B	0.28446	0.17698	0.31391	0.29320	0.29625	0.17969	-0.61216	-0.65722
GNE_A	0.58414	0.72462	1.25555	1.08503	1.27822	1.25123	1.32777	1.32193
GNE_B	-0.41453	0.23669	-0.45005	-0.27774	-1.25530	-1.23202	0.02183	0.23080
GRK4_A	-0.61933	-1.26370	0.09596	0.19903	-0.04101	-0.24485	0.29267	0.32398
GRK4_B	-3.61461	-3.61292	-5.53563	-5.28015	-2.76373	-4.10899	-4.97425	-5.24412
GRK5_A	0.15305	0.19311	-0.02443	-0.23553	-0.01550	-0.00522	-0.77223	-0.71742
GRK5_B	-1.79619	-1.79535	-1.45857	-1.04224	-1.47266	-1.12922	-1.04584	-1.26279
GRK6_A	-0.44712	-0.01362	0.45469	0.33328	-0.22957	-0.25527	0.62674	0.43914
GRK6_B	0.48405	0.69545	0.98724	0.94850	-0.75244	-0.67722	0.62962	0.71152
GSK3A_A	1.11899	1.31223	1.27746	1.26518	1.31345	1.39615	0.76091	1.25771
GSK3A_B	1.12435	1.53427	0.73156	0.69854	1.13893	1.19710	0.80355	1.09353
GSK3B_A	-1.71112	-2.06070	-1.39622	-0.36532	-0.21966	-0.11710	0.02210	0.08928
GSK3B_B	0.87663	0.99140	0.98127	0.91924	0.55417	0.88045	0.98420	1.09714
GUK1_A	-0.06154	-0.10281	-0.49178	-0.59482	0.49362	0.56052	0.99670	1.10233
GUK1_B	-0.61766	-0.66721	-0.64549	-0.89827	-1.02058	-0.80809	0.19428	0.30570
HAK_A	0.48814	0.43686	-0.46328	-0.42384	-0.28747	-0.12370	0.34588	0.48933
HAK_B	0.69618	0.78804	0.01547	0.37994	0.46735	0.30182	0.36415	0.51335
HCK_A	0.55311	0.36453	0.51506	0.17107	1.01964	1.12172	0.97106	0.99413
HCK_B	-1.01619	-1.08259	-0.64213	-1.01832	1.10295	1.15248	0.34641	0.36327
HGS_A	-0.13881	-0.06223	-0.91194	-1.18586	-0.67701	-0.90635	-0.18490	0.03396
HGS_B	0.71868	0.64600	0.45032	0.61612	0.43661	0.38356	0.68614	0.65159
HIPK1_A	-1.51487	-1.52906	0.10000	0.12250	1.08455	1.00157	-0.18316	-0.35928
HIPK1_B	-0.86480	-0.71278	-0.38439	-0.47382	-1.23136	-1.28830	-0.56331	-0.85918
HIPK2_A	-1.94538	-2.59134	0.68922	0.54266	-0.37060	-0.25896	-0.67578	-0.67560
HIPK2_B	0.95433	1.26770	0.90709	0.90925	1.01291	1.04318	0.91063	0.68787
HIPK3_A	-0.59642	-0.41067	0.08954	0.32346	-0.34074	-0.36032	-0.75416	-0.95239
HIPK3_B	0.56827	0.76785	-0.24222	-0.06712	0.17632	-0.04222	0.46321	0.62750
HK1_A	-3.97505	-4.08352	-3.14795	-3.26666	-3.91688	-4.17375	-5.61217	-5.79397
HK1_B	0.40080	0.72629	-0.89656	-0.70331	-0.50980	-0.62371	0.08091	0.04729
HK2_A	-0.74780	-0.77559	0.02349	-0.05430	-0.96821	-1.05250	-0.52995	-0.42352
HK2_B	-0.03273	-0.00912	0.73886	0.73782	0.01056	-0.25563	0.61806	1.10610
HK3_A	0.20464	0.26411	0.11831	0.02873	0.18594	0.10286	0.35471	0.12882
HK3_B	0.56879	0.84119	0.79063	0.66083	0.69248	0.61309	0.54831	0.42579
HSMDPKIN_A	0.33631	0.33203	-0.93333	-1.03808	-0.77697	-0.24553	-0.27182	-0.16336
HSMDPKIN_B	0.68165	0.64671	0.23230	0.30150	-0.06239	0.09754	0.31016	0.38488
HSPB8_A	-0.20906	0.31085	-0.09785	-0.01861	-0.06577	-0.28392	0.36819	0.15618
HSPB8_B	0.12474	0.03348	0.27035	0.06691	0.07264	-0.07412	0.38641	0.29313
HUNK_A	1.01531	0.89220	0.58847	0.51967	1.07028	1.08712	0.59998	0.63828
HUNK_B	-1.40513	-1.26958	-1.74600	-1.62917	-1.17187	-1.06808	-1.02666	-1.16299

Additional File 2: Z-score values for kinase siRNA library screen of Ewing's sarcoma cells (cont)

siRNA	TC-32_a	TC-32_b	TC-71_a	TC-71_b	SK-ES-1_a	SK-ES-1_b	RD-ES_a	RD-ES_b
IBTK_A	0.07249	-0.12889	-0.11693	-0.12300	-1.03070	-1.08621	-0.82995	-0.82687
IBTK_B	0.03981	-0.09382	0.41614	0.40389	0.31020	0.08927	0.01997	-0.08682
ICK_A	0.34691	0.44613	0.76266	0.71048	0.97388	0.76857	0.91074	0.61497
ICK_B	0.66939	0.73207	0.57334	0.30286	0.36845	0.51998	0.41658	0.32543
IHPK1_A	0.83026	1.06528	0.23492	0.36917	0.81831	0.93344	0.52051	0.38231
IHPK1_B	-0.13320	-0.24666	0.70762	0.66046	0.54463	0.57566	0.03605	-0.17459
IHPK2_A	0.08929	-0.00537	0.46253	0.13733	-0.00403	0.02537	0.15860	0.16588
IHPK2_B	-1.43057	-1.59597	0.20304	-0.04826	-0.29422	-0.47587	-0.67560	-0.72120
IHPK3_A	-1.28567	-0.94235	-0.43257	-0.27090	-0.08999	-0.03708	0.47790	0.47602
IHPK3_B	-0.80271	-0.29890	-0.22655	-0.12961	-0.17130	-0.11404	0.43513	0.37835
IKBKAP_A	0.42839	0.18693	0.19021	0.15217	-0.02161	0.12175	0.09763	0.05605
IKBKAP_B	0.60305	0.68103	0.31055	0.45653	-0.50484	-0.25642	-0.83459	-0.99050
IKBKE_A	-1.49185	-1.36150	-0.15902	-0.19297	-0.35296	-0.25103	-0.74356	-0.52977
IKBKE_B	0.22817	0.26614	0.20937	0.22794	0.02771	0.22143	0.36134	0.42207
IKBKG_A	-2.09287	-2.33197	-1.37093	-1.00321	-0.69786	-0.73793	-1.20132	-1.08269
IKBKG_B	0.17745	-0.03241	0.59993	0.64661	0.12728	-0.11273	-0.11772	-0.04049
ILK_A	-1.35593	-1.28489	-2.08064	-2.20024	-1.17343	-0.89080	-0.60930	-0.59434
ILK_B	0.44661	0.50639	0.83995	0.92096	0.77189	0.86332	0.50423	0.37422
ILKAP_A	-1.15762	-1.28405	0.12159	-0.10943	-0.49047	-0.51960	0.01330	-0.07231
ILKAP_B	-0.85244	-0.80699	0.44981	0.20693	0.41451	0.37199	0.48667	0.36420
IRAK1_A	1.01763	0.96674	0.55057	0.44636	0.15394	0.12392	0.32240	0.23451
IRAK1_B	-0.25364	-0.11524	0.06711	0.01571	0.36445	0.23668	-0.02533	0.06588
IRAK2_A	0.14161	0.22279	0.00928	-0.06727	0.57970	0.49982	0.59349	0.45822
IRAK2_B	-0.01569	-0.09314	-1.49260	-1.55016	-1.63887	-1.67562	-0.61542	-0.79209
IRAK3_A	0.25099	0.44448	-0.11076	-0.05071	-0.06627	0.00072	0.40413	0.42657
IRAK3_B	1.24628	0.91639	0.82863	0.82946	0.19495	0.14284	0.89017	0.94942
IRAK4_A	0.92726	1.12685	1.06336	1.01605	0.73313	0.75501	0.88573	0.80654
IRAK4_B	-0.01007	-0.05506	-0.02127	-0.10925	-0.13601	-0.00303	0.21716	0.11853
ITK_A	1.54669	1.54340	1.03821	1.11242	1.25607	1.35475	1.17336	1.25216
ITK_B	1.16254	1.16609	0.59835	0.53186	0.61310	0.30233	0.67599	0.93081
ITPK1_A	0.41824	0.53785	0.77219	0.63218	0.72266	0.69076	-0.11602	0.08599
ITPK1_B	0.95129	0.91802	0.84685	0.82661	1.01524	0.99340	0.31144	0.40919
ITPKA_A	-1.53885	-1.21272	-0.24890	-0.36260	-0.10275	-0.07450	-0.99285	-1.01538
ITPKA_B	-0.65092	-0.44020	-0.58458	-0.82436	-0.97112	-0.93198	-1.73688	-1.60906
ITPKB_A	0.43517	0.68762	0.94433	0.92371	0.91451	1.00880	1.08530	0.46293
ITPKB_B	0.85245	1.10831	0.36589	0.36491	0.77522	0.86628	0.53056	0.55200
ITPKC_A	-0.62516	-0.62641	-1.66382	-1.46448	-0.92954	-0.91856	-0.94689	-0.96402
ITPKC_B	0.83043	0.66938	0.86570	0.91542	0.67048	0.60102	0.35722	0.27850
JAK1_A	1.50932	1.52286	1.12011	1.20689	1.34333	1.38309	1.35163	1.31589
JAK1_B	-0.93397	-0.97706	-1.83294	-1.70757	-1.14784	-1.15493	-1.19815	-1.34577
JAK2_A	0.46504	0.40959	1.03623	1.08165	1.06027	1.05846	0.92723	0.82961
JAK2_B	0.67391	0.76590	0.68437	0.74241	0.74253	0.64427	0.52399	0.42100
JAK3_A	1.12893	1.30460	1.13906	1.12958	1.15579	1.23366	1.14169	1.16251
JAK3_B	-0.53672	-0.75080	-0.04277	0.06296	0.27348	0.39090	0.34334	0.39813
KDR_A	1.35280	1.28061	1.09191	1.06032	1.30386	1.13740	0.94388	1.02141
KDR_B	-0.84516	-0.97368	-2.14640	-2.01490	-2.57391	-2.54575	-1.07101	-0.91004
KHK_A	-1.00200	-1.21933	-0.69421	-0.65685	-0.09390	-0.12018	-0.03764	-0.20351
KHK_B	1.02381	0.79259	0.87302	0.58662	0.49675	0.56304	0.44508	0.27444
KIAA1446_A	0.07683	0.05951	0.64620	0.61547	0.41973	0.53914	0.40777	0.17590
KIAA1446_B	0.43110	0.26847	0.21641	0.23440	0.88073	0.87000	0.42139	0.20078
KIAA1804_A	-0.21502	-0.30055	-1.40093	-0.94313	-0.75614	-0.71297	0.16698	0.12176
KIAA1804_B	0.43319	0.11968	0.99453	0.82521	0.82100	0.70416	0.34461	0.73513
KIDINS220_A	1.26632	1.29660	1.03589	1.01611	1.32934	1.26094	1.06623	1.10050
KIDINS220_B	0.05368	-0.01663	0.86742	0.79989	0.69800	0.63154	0.83230	0.83914
KIP2_A	-0.26686	-0.21464	-0.01261	0.09275	0.44175	0.43563	0.37452	0.36609
KIP2_B	-0.33546	0.01983	0.41654	0.27253	0.76531	0.98253	-0.35411	-0.13762
KIS_A	0.38677	0.15734	0.44314	0.41022	0.57421	0.89861	0.50802	0.53134
KIS_B	0.38494	0.44136	0.28333	0.05926	-0.24318	-0.21683	0.82165	0.61649
KIT_A	0.40113	0.32950	0.52775	0.50530	0.12511	0.30090	0.30103	0.22850
KIT_B	0.87785	1.00823	0.99744	0.82361	0.02333	-0.03744	0.74149	0.67924
KSR_A	1.26054	1.07535	0.84272	0.91378	0.97299	0.98434	0.66810	0.84492
KSR_B	-0.46021	-0.70504	-0.59054	-0.71766	-0.60323	-0.71147	-0.03367	0.70498
KSR2_A	0.63541	0.57259	1.04091	1.10643	0.67561	0.67015	0.08713	-0.03042
KSR2_B	-0.74819	-0.99441	-0.14297	-0.15998	-0.56286	-0.41167	-0.59593	-0.60336
LATA_A	0.85273	0.82204	0.75465	0.79677	0.69319	0.86191	0.61067	0.65289
LATS1_B	0.73322	0.68023	1.07819	0.93921	1.01996	1.15686	1.02036	1.03037
LATS2_A	0.21978	0.36150	0.14303	0.31403	0.78296	1.10210	0.96938	0.93884
LATS2_B	-0.78312	-0.75793	-0.46699	-0.44242	-1.75667	-1.75363	-1.28119	-1.39441
LIM_A	-0.60459	-0.51203	0.19129	0.07776	0.76308	0.67265	0.46861	0.38173
LIM_B	0.44020	0.26314	0.88108	0.57529	0.63779	0.50875	0.12681	0.24457
LIMK1_A	0.60178	0.65286	-0.07180	-0.14131	0.20543	0.22157	0.19242	0.13065
LIMK1_B	0.70903	0.66584	0.19182	0.18250	0.17618	0.34903	0.00846	0.04816
LIMK2_A	0.73988	0.55275	0.56088	0.54046	0.99452	0.31407	0.44073	0.40818
LIMK2_B	0.64487	0.58192	0.23884	0.12657	-0.25680	-0.52842	0.42199	0.36883
LMTK2_A	1.19710	0.93041	0.89846	0.98661	1.18067	1.07566	0.47672	0.47127
LMTK2_B	0.49608	0.29933	0.51162	0.43596	0.58500	0.43431	0.78925	0.73056
LOC161635_A	0.87311	0.69053	1.02521	1.07860	1.12778	1.04614	0.94432	1.03686
LOC161635_B	1.26876	1.23816	0.89267	0.92264	1.02087	0.91178	0.67789	0.73563
LOC375449_A	0.69645	0.61721	0.88683	0.92190	0.33966	0.36327	0.15699	0.24842
LOC375449_B	0.21897	0.25144	0.68804	0.81356	0.72568	0.80638	0.64610	0.60715
LOC388226_A	0.05497	-0.23586	0.50971	0.57536	0.45888	0.64348	0.32670	0.35235
LOC388226_B	0.79510	0.86658	-0.12251	-0.13655	0.39819	0.45599	0.44400	0.41197
LOC55971_A	-1.40614	-1.16349	-1.08034	-1.65281	-0.98182	-1.04674	-0.64627	-0.35676
LOC55971_B	-0.50043	-0.59845	-0.57163	-0.64520	-0.97014	-1.01356	-1.31217	-1.31013
LOC91807_A	1.34953	1.48280	1.15491	1.10304	1.24224	1.02228	0.82673	0.99752
LOC91807_B	-0.10110	-0.20515	0.66428	0.44727	0.21317	0.31875	0.28977	0.31135
LTK_A	1.06006	1.16424	1.08330	1.07614	1.16849	1.07467	1.04122	1.20505
LTK_B	0.30242	0.20088	0.58673	0.46935	0.60705	0.73592	0.81321	0.98695
LYK5_A	-0.46925	-0.54987	0.36810	0.35293	0.24011	0.17433	0.07088	0.13828
LYK5_B	0.69574	0.31898	0.39528	0.39526	0.05700	0.14908	0.06619	0.05696
LYN_A	0.78995	0.64891	0.73502	0.80302	0.58916	0.55500	1.09837	0.97934
LYN_B	0.54294	0.42159	0.05005	0.24461	0.21887	0.21340	0.73263	0.54999
MADD_A	0.17849	0.39968	0.64087	0.61896	0.32489	0.16052	0.30286	0.31939
MADD_B	1.36562	1.30563	0.96539	1.03144	1.05582	0.99058	1.02069	0.96029
MAGI1_A	-0.02006	-0.12256	0.27031	0.34002	0.53462	0.49137	0.11820	0.28297
MAGI1_B	-1.51496	-1.54072	-1.47520	-1.44744	-1.39752	-1.31058	-2.04023	-1.81326
MAGI-3_A	0.57215	0.63502	0.43100	0.35480	0.51386	0.44145	-0.48037	-0.47580
MAGI-3_B	0.81132	0.67097	1.04891	1.07653	1.09457	1.01479	0.72299	0.44336
MAK_A	1.06975	0.90544	0.77953	0.79817	1.12755	1.16191	0.78069	0.80268
MAK_B	1.34215	0.99610	1.05352	1.10396	0.94247	1.01204	0.39496	0.39119
MAP2K1_A	-2.66658	-2.67218	-5.05864	-5.08880	-3.00261	-2.39363	-1.96838	-2.35962
MAP2K1_B	0.21074	0.23864	-0.21154	-0.32344	-0.62034	-0.55602	-0.60267	-1.03192
MAP2K1IP1_A	0.54360	0.74446	0.10074	0.53063	0.74313	0.53926	0.33314	0.09688
MAP2K1IP1_B	-1.11197	-1.14525	0.00114	0.04576	0.93013	0.80675	0.95769	0.83931
MAP2K2_A	0.20525	0.04383	-0.03074	-0.17771	-0.68906	-0.70461	-0.37859	-0.35047
MAP2K2_B	-1.00154	-1.02127	-1.73252	-2.02836	-0.52501	-0.67924	-0.51520	-0.54761
MAP2K3_A	-1.49506	-1.64170	-1.17165	-1.13220	-0.15372	-0.15469	0.01140	0.02675
MAP2K3_B	-0.04060	0.05700	0.29342	0.13063	0.43045	0.41107	0.34825	0.38546

siRNA	TC-32_a	TC-32_b	TC-71_a	TC-71_b	SK-ES-1_a	SK-ES-1_b	RD-ES_a	RD-ES_b
MAP2K4_A	-0.14311	-0.03627	0.61218	0.43118	0.10798	0.21811	0.42521	0.62976
MAP2K4_B	0.43511	0.53679	0.53924	0.47834	0.83845	0.73188	1.04980	1.06386
MAP2K5_A	-0.03831	0.06306	-0.77631	-0.79700	-0.17948	-0.14878	-3.02563	-2.85121
MAP2K5_B	0.36419	0.41635	0.15006	0.30727	-0.81848	-0.86367	-0.35801	-0.26931
MAP2K6_A	-0.62408	-0.64009	-0.38985	-0.28003	-0.08108	-0.20078	-1.06993	-1.10081
MAP2K6_B	0.61378	0.66578	0.41039	0.43702	0.28634	0.13920	-0.02813	0.14933
MAP2K7_A	-0.86934	-0.97239	-1.05434	-0.89214	-1.00901	-1.05433	-0.18213	-0.02355
MAP2K7_B	-2.60933	-2.37084	-0.66762	-0.30631	-0.99979	-0.87442	-0.47381	-0.62541
MAP3K1_A	-0.45379	-0.58540	-1.73125	-1.77344	-1.25077	-1.26390	-2.70421	-2.55558
MAP3K1_B	0.64579	0.64180	0.42907	0.44622	1.18157	1.10854	0.80719	0.74136
MAP3K10_A	1.37404	1.47669	0.89260	1.14160	1.44083	1.48543	1.10412	1.11435
MAP3K10_B	0.38867	0.32770	-0.29670	-0.19287	0.47003	0.38436	0.55305	0.46538
MAP3K11_A	1.29449	1.46529	1.13902	1.15691	1.25246	1.36385	1.14236	1.07049
MAP3K11_B	-0.77778	-0.88297	-1.37119	-1.46762	-0.38644	-0.34258	-0.55005	-0.63462
MAP3K13_A	0.72496	0.78874	0.56995	0.65859	1.09437	0.90895	0.66044	0.71443
MAP3K13_B	0.90645	0.71645	0.27116	0.23135	0.63849	0.53515	0.70981	0.63026
MAP3K2_A	0.64672	0.74983	0.47622	0.65570	-0.26278	-0.06114	0.09571	0.01811
MAP3K2_B	-0.55835	-0.52585	-0.19070	-0.12452	-0.67007	-0.44125	-1.02343	-0.93449
MAP3K3_A	0.43313	0.53683	0.53964	0.47106	0.40182	0.33396	0.53880	0.54973
MAP3K3_B	-0.10024	-0.24579	0.14302	0.04064	0.79902	0.83156	-0.10975	-0.26969
MAP3K4_A	0.43458	0.33951	0.16509	0.08038	-0.73152	-0.84308	-0.54819	-0.55012
MAP3K4_B	0.92278	0.80218	0.67406	0.84450	0.87057	0.83309	0.78072	0.63143
MAP3K5_A	-1.08864	-1.11942	-0.51036	-0.44998	-0.49007	-0.52488	-0.21018	-0.18017
MAP3K5_B	0.80213	0.92744	0.63963	0.76344	0.55533	0.60137	0.61793	0.79318
MAP3K6_A	-1.51388	-1.34103	0.12996	0.55579	-0.94432	-0.92209	-0.82834	-0.43956
MAP3K6_B	0.55577	0.64536	-0.11884	0.04699	-0.81304	-0.89632	-1.49753	-1.27368
MAP3K7_A	-0.24738	-0.07993	0.69684	0.84075	-0.02893	-0.10343	0.00708	-0.19014
MAP3K7_B	-0.08855	-0.12463	-1.53448	-1.66583	-1.60161	-1.62243	-1.23335	-1.21316
MAP3K7IP1_A	-0.67590	-0.56952	-1.25075	-1.18009	-0.82257	-0.70963	-0.66242	-0.74832
MAP3K7IP1_B	0.47431	0.53351	0.72066	0.45318	0.76815	0.74364	0.63157	0.49972
MAP3K7IP2_A	-2.13320	-2.35374	-0.52305	-0.38807	-1.44822	-1.16382	-1.05892	-0.99132
MAP3K7IP2_B	0.83837	0.94909	1.15981	1.21063	0.98535	1.13995	1.14709	1.06888
MAP3K9_A	1.47608	1.40434	1.11317	1.14104	1.33693	1.52466	1.10138	1.15812
MAP3K9_B	1.01697	0.71660	1.00300	1.03874	1.15875	1.19926	0.64594	0.73004
MAP4K1_A	-2.05338	-2.06864	-2.23757	-1.78136	-2.53744	-2.36924	-3.88397	-3.80200
MAP4K1_B	0.72114	0.65710	0.04710	0.02584	0.18896	0.13917	0.11849	0.25546
MAP4K2_A	0.07536	-0.13787	-0.63190	-0.57723	-0.29967	-0.35602	0.01305	0.05484
MAP4K2_B	-0.52308	-0.64775	0.44510	0.49124	0.48559	-0.48021	0.00522	0.01266
MAP4K3_A	0.41614	0.17605	0.45883	0.62657	-0.25210	-0.27026	0.49250	0.27895
MAP4K3_B	-0.47706	-0.62330	0.13581	0.27842	-0.46712	-0.32462	-0.30447	-0.76028
MAP4K4_A	-1.02602	-0.90141	-0.14363	-0.22767	-0.35874	-0.33657	-0.30859	-0.06305
MAP4K4_B	-1.75145	-1.60959	-0.47850	-0.66547	-0.51210	-0.32846	-0.41995	-0.32780
MAP4K5_A	0.93186	0.84001	0.62048	0.68643	0.60917	0.69338	0.46038	0.29213
MAP4K5_B	-1.27998	-1.50213	-0.25472	-0.07360	-1.20931	-1.06418	-0.15966	-0.25865
MAPK1_A	1.37645	1.46878	1.33014	1.13553	1.34022	1.39161	1.01050	1.08148
MAPK1_B	0.61025	0.70908	0.78395	0.78877	1.01118	0.95730	0.84755	1.02483
MAPK10_A	1.27145	1.22811	1.27584	1.17817	1.32688	1.34133	0.65311	1.11225
MAPK10_B	1.23430	0.99668	1.00931	0.74406	1.02890	0.98809	0.81955	0.86774
MAPK11_A	-2.58749	-2.35856	-2.50905	-2.68682	-1.74276	-1.62432	-1.31228	-1.36708
MAPK11_B	1.16502	1.07388	0.92583	0.79121	1.11382	0.86521	0.87364	0.79046
MAPK12_A	-2.07072	-1.98540	-3.67337	-3.74326	-2.33911	-2.38534	-3.75546	-3.87022
MAPK12_B	0.80798	0.87143	0.88580	0.93917	0.40544	0.36202	0.67304	0.65773
MAPK13_A	0.09842	0.39594	0.17713	0.15544	-0.47631	-0.61400	-0.05556	-0.16235
MAPK13_B	-0.98087	-0.65144	-0.82418	-0.93109	-2.35967	-1.88591	-1.87552	-1.80568
MAPK14_A	-0.07772	-0.01823	-0.26596	-0.21193	0.45325	0.32594	0.35371	0.33716
MAPK14_B	0.38155	0.25341	-0.48035	-0.77248	0.12347	0.04122	-0.37174	-0.27703
MAPK3_A	0.63806	1.38771	0.47870	0.38815	0.96299	0.81017	0.72372	1.07640
MAPK3_B	0.69587	0.79593	0.82992	0.71642	0.83951	0.83028	0.78176	0.72135
MAPK4_A	0.35085	0.51902	0.38854	0.19717	0.69213	0.84487	-0.21092	-0.06175
MAPK4_B	0.32675	0.41407	-0.06914	-0.13251	-0.50703	-0.53411	-0.29736	-0.54346
MAPK6_A	0.43575	0.25467	-0.49753	-0.58011	-0.62888	-0.59896	-0.06690	-0.13765
MAPK6_B	0.84874	0.59975	0.30311	0.32594	0.38884	0.53136	0.28877	0.23660
MAPK7_A	0.37605	0.61536	0.24251	0.26222	-0.77475	-0.67487	-0.12619	0.00287
MAPK7_B	0.24941	-0.19344	-0.05766	-0.22848	0.09212	-0.04338	-0.19160	-0.34732
MAPK8_A	0.81545	0.82933	0.77456	0.90561	0.88321	0.78000	1.01225	0.98881
MAPK8_B	0.44053	0.70572	0.31785	0.48541	0.50704	0.47039	0.78716	0.92953
MAPK8IP1_A	-0.09266	0.03054	-1.40572	-1.46908	-1.07567	-0.98963	-0.94497	-0.96363
MAPK8IP1_B	0.26766	-0.06449	0.42756	0.54546	-0.43373	-0.38515	-0.34766	-0.33463
MAPK8IP2_A	-0.89654	-0.88620	0.07849	0.05670	-1.00866	-0.93394	-1.46487	-1.45466
MAPK8IP2_B	0.82968	0.86171	1.05215	0.99569	0.66451	0.46038	0.50241	0.46820
MAPK9_A	-0.23572	-0.34849	-0.19232	-0.22336	-0.05400	-0.03483	-0.21545	-0.31452
MAPK9_B	1.14573	1.21793	1.01893	0.96895	1.26596	1.18958	0.96949	0.95030
MAPKAP1_A	0.70743	0.78014	0.61386	0.72539	0.05155	0.00926	-0.27390	-0.18849
MAPKAP1_B	-0.55806	-0.64788	0.01505	-0.00828	-0.20145	-0.38878	-0.99235	-1.04067
MAPKAPK2_A	-2.21274	-2.34642	-1.67035	-1.73692	0.13490	0.13836	0.20252	0.02838
MAPKAPK2_B	0.44500	0.37706	0.03161	-0.02641	-0.36975	-0.37060	0.31300	0.22970
MAPKAPK3_A	0.09200	0.14008	-0.47562	-0.26021	0.39141	0.15129	0.67232	0.75646
MAPKAPK3_B	-0.13146	-0.11274	-1.86849	-1.25724	0.24204	0.07233	0.40387	0.53212
MAPKBP1_A	0.96257	0.93963	0.81578	0.54848	0.64756	0.65256	0.66266	0.60036
MAPKBP1_B	0.74267	0.65595	0.93616	0.89768	0.53019	0.53646	0.67388	0.57586
MARCKS_A	-0.91644	-1.35377	-0.80355	-0.71105	-0.99091	-1.77308	-0.76491	-0.76192
MARCKS_B	-1.46491	-1.64905	-0.36226	-0.24286	-0.03732	-0.34313	-1.16669	-0.51910
MARK1_A	0.67123	0.59036	0.22149	0.38024	0.66490	0.73008	0.52759	0.40244
MARK1_B	-0.16647	-0.43277	-0.53902	-0.21095	-0.70109	-0.39278	-0.20455	-0.31440
MARK2_A	0.52159	0.22172	0.09041	-0.50398	0.40949	0.58781	0.59385	0.65205
MARK2_B	1.37307	1.22235	0.82611	0.57046	1.01962	1.26980	1.10691	1.08597
MARK3_A	1.33393	1.32058	1.17401	1.33949	1.13268	1.27306	1.22550	1.20121
MARK3_B	1.15652	1.15253	0.33201	0.42459	0.57740	0.44036	0.93346	0.89874
MARK4_A	0.12145	0.44813	0.40032	0.02488	0.49176	0.73113	0.49575	0.55495
MARK4_B	0.20696	0.07612	0.03316	-0.07303	0.96116	1.15784	0.80500	0.77288
MAST1_A	-0.77514	-0.72365	-1.26431	-1.24277	-0.49518	-0.16218	-1.20963	-1.19567
MAST1_B	-2.77081	-3.35968	-1.81928	-1.69818	-1.67082	-1.60056	-1.82913	-1.95350
MAST2_A	-0.26622	-1.76345	-2.49486	-3.18142	-1.54321	-1.21825	-0.72640	-0.78704
MAST2_B	0.09067	0.04796	0.25292	0.27892	0.00988	-0.01571	0.35096	0.43586
MATK_A	0.62668	0.39551	0.09119	0.21435	0.51209	0.52390	0.41555	0.49090
MATK_B	0.52236	0.37974	0.56588	0.52118	0.41141	0.40436	0.28607	0.17948
MELK_A	0.25433	0.27328	0.04044	0.18072	-0.12267	-0.66305	-0.89454	-0.55841
MELK_B	0.84999	0.87292	0.50912	0.48392	1.13452	0.90967	0.28759	0.37831
MERTK_A	-0.48840	-0.60113	-1.08532	-1.16519	-1.07012	-0.80370	-1.31837	-1.30369
MERTK_B	0.45069	-0.22013	0.51359	0.54223	0.05147	-0.01844	0.23803	0.24531
MET_A	0.59660	0.60192	0.27466	0.23552	0.00846	-0.01440	0.65657	0.62795
MET_B	0.82075	0.84160	1.09272	1.25004	1.11379	1.10665	1.05948	0.97993
MGC4796_A	-1.61801	-1.80704	-0.42184	-0.45976	-0.88056	-1.04703	-0.36913	-0.36756
MGC4796_B	-1.52743	-1.78413	-1.34079	-1.07436	-1.12652	-1.11305	-0.86948	-0.82077
MKNK1_A	-0.56295	-0.91197	0.03490	0.01506	-0.41073	-0.35268	0.14900	-0.04752
MKNK1_B	-0.49522	-1.60627	-0.48046	-0.76073	-1.79220	-1.71524	-0.89950	-0.86814
MKNK2_A	0.86834	0.89214	0.72387	0.77319	0.90570	1.03458	0.54802	0.58781
MKNK2_B	-0.05018	-0.02548	-0.45237	-0.31670	-0.52657	-0.42721	-0.48496	-0.65119

Additional File 2: Z-score values for kinase siRNA library screen of Ewing's sarcoma cells (cont)

siRNA	TC-32_a	TC-32_b	TC-71_a	TC-71_b	SK-ES-1_a	SK-ES-1_b	RD-ES_a	RD-ES_b
MK-STYX_A	-1.99800	-1.77542	-1.49910	-1.65761	-2.00015	-2.18751	-3.07840	-3.24853
MK-STYX_B	0.48836	0.34927	0.71070	0.69265	0.63973	0.57842	0.27684	0.18180
MOS_A	0.80442	0.75642	0.27002	0.68321	1.17162	1.01763	0.52603	0.71734
MOS_B	0.64238	0.71302	0.50609	0.59478	0.22451	0.19125	0.06470	0.09374
MST1R_A	0.38672	0.57387	0.43570	0.51411	0.42454	0.36852	0.55646	0.64907
MST1R_B	0.77896	0.79670	0.12730	0.26764	0.52858	0.48895	0.26903	0.20857
MST4_A	0.66812	0.65692	0.29676	0.26472	-0.11847	-0.01670	0.16089	0.17828
MST4_B	1.12524	0.92195	0.71491	0.54630	1.08346	0.95162	0.31514	0.37307
MVK_A	0.20485	-0.08932	0.73613	0.38279	0.38384	0.37517	0.74981	0.91496
MVK_B	0.03973	-0.09349	-1.44367	-1.48763	-0.94742	-1.05737	-1.15957	-0.94962
MYLK_A	1.20698	1.03037	0.83954	0.97553	0.47357	0.58927	0.79343	0.65149
MYLK_B	-0.14395	-0.19680	-0.18955	0.08971	0.54107	-0.43339	0.32342	0.25081
MYLK2_A	0.26869	0.45395	0.74070	0.69144	0.18346	0.22510	0.01769	0.05779
MYLK2_B	1.20537	1.19050	0.32433	0.46939	1.01675	1.03129	0.71071	0.96295
NAGK_A	-1.01219	-0.80073	-0.76518	-0.69842	-1.28082	-1.26410	-1.13732	-1.12305
NAGK_B	-0.42344	-0.54874	-1.75544	-2.09107	-0.64760	-0.72621	-0.74581	-0.86774
NEK1_A	-1.02852	-0.77497	0.20228	0.16861	-0.74345	-0.69997	0.09859	-0.12114
NEK1_B	0.88067	0.86873	0.74848	0.71858	0.48388	0.72558	0.77282	0.42925
NEK11_A	0.73818	0.68965	0.92063	0.88513	0.73805	0.83604	0.24892	0.16951
NEK11_B	-1.37543	-1.76535	-0.43266	-0.51904	-1.26331	-1.24479	-0.19274	-0.48552
NEK2_A	0.42730	0.52448	0.06662	0.15115	0.35448	0.48397	0.59417	0.89597
NEK2_B	0.55331	0.81411	0.45572	0.64933	0.09657	0.37921	0.94325	0.94142
NEK3_A	-2.39169	-2.55468	-4.37074	-3.34780	-2.10575	-2.24684	-4.22688	-4.19102
NEK3_B	1.35025	1.38241	1.15302	1.08194	1.54483	1.40773	1.08946	1.19881
NEK4_A	-0.00564	-0.15293	0.15682	-0.08874	-1.51982	-1.46300	-0.91002	-0.58833
NEK4_B	1.12036	1.23984	1.14172	1.04244	1.46563	1.28027	0.89386	0.92755
NEK6_A	0.68959	0.71321	0.14529	0.16207	0.35687	0.27699	0.82444	0.67915
NEK6_B	0.86762	0.89118	0.33392	0.00942	0.50016	0.54989	0.70473	0.51625
NEK7_A	0.47069	0.50814	0.27995	-0.26886	0.57981	0.57769	0.99144	0.74937
NEK7_B	-0.74512	-0.78651	-0.17446	-0.30459	-1.19486	-1.23911	-0.20811	-0.59478
NEK8_A	-0.59580	-0.34683	-0.05951	0.16890	0.47153	0.66030	-0.60825	-0.60041
NEK8_B	-1.45044	-1.65461	-1.45179	-1.47188	0.71937	0.70803	0.42559	0.37454
NEK9_A	-3.22990	-1.49934	-4.11148	-3.47465	-2.09111	-2.96414	-2.19894	-2.28717
NEK9_B	0.39757	0.41995	0.46121	0.44637	0.52522	0.55003	0.60564	0.83801
NIPA_A	0.29438	0.11821	0.16983	0.34387	0.52854	0.59796	-0.48275	-0.56345
NIPA_B	-1.24184	-1.11239	-0.49030	-0.55168	-1.20557	-1.12797	-0.39367	-0.42486
NJMU-R1_A	-0.94903	-0.95529	-1.64932	-1.57249	0.06205	-0.34515	0.35599	0.35496
NJMU-R1_B	1.09716	0.92075	0.08736	-1.53207	0.51877	0.20614	0.39855	0.33817
NLK_A	-1.12997	-0.73165	-1.85427	-1.72811	-1.60536	-1.49758	-2.54539	-3.05968
NLK_B	-0.40330	-0.55593	0.10815	0.03315	0.22796	0.32384	0.05273	0.00124
NME1_A	1.22455	1.21293	0.90695	0.97682	0.80655	0.71784	0.61665	0.74566
NME1_B	-0.02923	0.15300	0.25169	0.26523	1.01845	0.96266	-0.49133	-0.47913
NME2_A	-1.32082	-1.29836	-0.63998	-0.27357	0.86747	0.56720	-0.79449	0.82538
NME2_B	-0.32131	-0.40655	-0.01533	0.10048	-0.13426	-0.45069	0.27272	0.37663
NME3_A	-3.89748	-3.19996	-2.39187	-2.56734	-2.54342	-2.55492	-1.14684	-1.25904
NME3_B	0.26454	0.62922	0.32627	0.26608	0.45404	0.31980	0.01199	-0.02621
NME4_A	0.78298	0.89901	1.00910	0.82308	0.79449	0.61830	0.05635	0.26052
NME4_B	-1.02482	-0.99324	0.77419	0.57265	0.18834	0.06171	0.10027	0.09524
NME5_A	0.31120	-0.38593	0.50486	0.35399	-0.56858	-0.60002	0.35909	0.23449
NME5_B	-0.24664	-0.36382	0.50519	0.27183	0.30962	0.07600	0.39930	0.28911
NME6_A	-1.83506	-1.68784	-1.37296	-0.82663	-0.90444	-0.80916	-0.32448	-0.96750
NME6_B	0.93334	1.08951	0.84433	0.99951	0.97553	0.93131	0.82190	0.80565
NME7_A	-1.96014	-1.85721	-2.04045	-2.09747	-1.07643	-0.87914	-1.07929	-0.89482
NME7_B	0.16701	0.15747	0.37913	0.42055	0.85810	0.61684	0.42413	0.33852
NRBP_A	0.61086	0.41315	-0.36080	-0.13329	0.41029	0.40589	0.57950	0.43635
NRBP_B	-0.34102	-0.57923	-1.71538	-1.77167	-1.33533	-1.42635	-0.76652	-0.83964
NRGN_A	-1.38570	-1.35481	-2.17243	-2.67590	-0.49357	-0.51405	-0.05450	0.00434
NRGN_B	-0.30069	-1.11100	-1.29555	-1.36029	-1.18761	-1.35533	0.38189	0.52006
NTRK3_A	0.24574	0.18175	0.27653	0.07472	0.50114	0.51993	0.49001	0.40401
NTRK3_B	-1.93884	-1.93417	-2.14388	-2.07241	-2.17359	-2.01484	-1.65708	-1.77179
NUCKS_A	0.94381	0.92584	0.97102	1.17062	0.74867	0.63275	0.47908	0.39058
NUCKS_B	0.87511	0.88672	0.66782	0.71059	0.86746	0.66412	0.54008	0.56468
NYD-SP25_A	-3.00535	-3.00353	-3.38141	-3.28428	-0.62952	-0.74828	-1.07655	-1.02713
NYD-SP25_B	-0.25931	-0.40198	0.49095	0.51472	0.74602	0.60733	0.95103	0.86978
PACSIN2_A	0.23150	0.39567	-0.47497	-0.71787	-0.41554	-0.60450	0.01274	-0.17866
PACSIN2_B	0.27516	0.06530	0.76197	0.83910	0.85110	0.60262	0.44105	0.27831
PACSIN3_A	-0.44540	-0.43277	-0.14294	-0.03694	-0.12975	-0.17596	-0.35899	-0.41502
PACSIN3_B	0.42015	0.53459	0.32852	0.35157	0.30292	0.24024	0.39819	0.50539
PAK1_A	-0.20570	-0.16474	-0.87309	-1.01396	-2.03885	-1.87653	-1.08166	-1.35373
PAK1_B	0.38417	0.38040	0.32049	0.26058	0.27707	0.16435	-0.70123	-0.64830
PAK2_A	0.07976	0.37240	0.62407	0.59347	0.84188	0.90984	1.02580	1.04961
PAK2_B	0.33119	0.04956	0.46244	0.80792	0.61901	0.69810	0.43650	0.39817
PAK3_A	0.81621	0.97819	0.23046	0.02830	-0.13140	-0.17309	0.28077	0.53519
PAK3_B	0.11084	0.06299	0.69313	0.45182	0.53855	0.45837	0.57452	0.66220
PAK4_A	0.82948	0.80320	-0.45465	-0.02749	-1.00013	-1.04761	0.00026	-0.15239
PAK4_B	-0.61630	-0.91825	0.56249	0.48488	-0.75325	-0.70699	-1.83179	-1.75705
PAK7_A	-3.04314	-3.19893	0.32349	0.34457	-0.48449	-0.28731	0.23872	0.12801
PAK7_B	0.37411	0.52384	0.85790	0.90834	0.69415	0.81904	0.44257	0.58671
PANK1_A	-0.65459	-0.67438	-0.66148	-0.80256	-1.18872	-1.22605	-3.29829	-1.37901
PANK1_B	-0.13979	-0.40122	0.08729	-0.09428	-0.51240	-0.45709	-0.04409	0.04453
PANK2_A	-0.26135	-0.17955	-1.25146	-1.16956	0.37223	0.37722	-0.44915	-0.69586
PANK2_B	-0.47591	-0.43151	0.04456	0.18139	-0.41319	-0.52968	-0.28687	-0.39372
PANK3_A	1.04121	0.92001	1.19293	0.84749	0.96103	1.05389	0.81931	0.85796
PANK3_B	0.07222	-0.13065	-0.10979	-0.49482	0.44467	-0.38550	-0.33342	-0.18971
PANK4_A	0.86633	0.74802	0.71482	0.62151	0.53551	0.33911	0.82098	0.57625
PANK4_B	-0.41818	-0.28805	0.54605	0.43503	1.02197	0.79960	0.65441	0.31787
PASK_A	1.09715	1.09505	1.08489	1.13081	0.93717	1.20306	1.00342	0.90778
PASK_B	0.38836	0.48660	0.47013	0.61150	0.02589	0.10295	0.42591	0.39984
PBK_A	-1.08915	-2.43502	-0.13266	-0.39078	-1.39484	-1.72744	-0.68768	-0.65142
PBK_B	0.67649	0.81168	-0.05504	0.11370	-0.78701	0.68321	0.53121	0.72055
PCTK1_A	0.03543	-0.24979	0.35230	0.23081	0.65035	0.73457	0.66009	0.66224
PCTK1_B	1.25505	1.21008	0.90162	0.49672	1.16059	1.17815	0.85813	0.98922
PCTK2_A	0.58006	0.54887	0.23867	0.66641	0.66236	0.50994	0.85686	0.58446
PCTK2_B	1.17680	1.05226	0.86626	0.78380	1.22067	1.09182	0.89984	0.79055
PCTK3_A	-2.26199	-2.09951	-4.22028	-3.64115	-0.97688	-0.81777	-0.49250	-0.62864
PCTK3_B	0.88805	1.13543	0.87691	1.02503	0.81262	0.50343	0.84301	0.70184
PDGFRA_A	0.72939	0.57671	-0.07893	0.13778	-0.12472	-0.31611	0.16905	0.18314
PDGFRA_B	-0.93738	-0.57459	-0.24611	-0.24577	-2.68339	-2.49801	-3.13158	-3.06501
PDGFRB_A	0.89368	0.86312	0.58197	0.67041	0.75072	0.85501	0.73434	0.77223
PDGFRB_B	-1.03318	-1.19960	0.04567	0.22757	-0.15364	-0.11370	0.16765	0.29309
PDIK1L_A	0.78332	0.72230	0.84339	0.95326	0.70382	0.74029	0.42283	0.51890
PDIK1L_B	-2.49223	-2.04401	-0.42782	-0.61288	-1.95426	-2.32377	-3.91164	-4.00018
PDK1_A	-1.61529	-0.29601	-2.93129	-2.34219	-2.01763	-1.90250	-1.39012	-1.45053
PDK1_B	1.20425	0.98858	0.62639	0.23148	0.85690	0.85488	0.82670	0.87313
PDK2_A	0.70444	0.58242	0.30008	0.17331	0.02355	0.09035	0.39319	0.32596
PDK2_B	-0.13675	-0.16870	0.59519	0.43004	0.62640	0.83532	0.04255	0.05526
PDK3_A	1.43476	1.56041	0.60641	0.70977	1.30329	1.36993	1.04102	1.22903
PDK3_B	0.50415	0.79508	-0.05400	-0.51215	-0.62182	-0.51627	0.69793	0.75290
PDK4_A	0.52254	0.35894	0.59625	0.48560	0.72009	0.85094	0.77117	0.82724
PDK4_B	-0.82230	-1.00310	-1.88738	-2.02252	-2.43935	-1.99084	-1.59045	-1.61757
PDPK1_A	-2.29275	-2.15094	-0.97090	-1.04647	-1.40497	-1.41934	-1.16444	-1.11799
PDPK1_B	0.77997	0.78592	0.49914	0.64650	0.87052	0.95248	0.51915	0.49334
PDXK_A	-1.85474	-1.57099	-1.18284	-1.01921	-1.30343	-0.49751	0.15040	0.47483
PDXK_B	-0.26327	-0.04093	-0.36070	-0.27273	-0.89115	-0.90241	0.10358	0.15641
PFKFB2_A	-0.37539	-0.06082	0.35351	0.21441	-1.18233	-0.94289	0.30901	0.02289
PFKFB2_B	-0.89620	-0.89364	-1.86517	-2.02101	-0.28830	-0.37245	-0.07269	-0.07187
PFKFB3_A	0.55213	0.61938	0.38883	0.26896	0.66910	0.76281	0.98118	0.99585
PFKFB3_B	1.07010	1.29878	0.72165	0.76505	0.57689	0.61715	1.02414	0.90052
PFKM_A	-0.30489	-0.73612	-1.73579	-1.21220	-0.89722	-0.63003	-0.61331	-0.47275
PFKM_B	-0.27431	-0.47433	-0.07945	0.04055	0.79143	0.91161	-0.39048	-0.71122
PFTK1_A	0.15925	0.32938	-0.09588	0.13063	0.19639	0.15500	0.38323	0.44014
PFTK1_B	-0.28018	-0.26156	-0.50471	-0.68288	-1.17451	-1.04744	-0.71399	-0.92742
PGK1_A	0.22179	-0.05319	-0.28978	-0.25888	-0.31497	-0.29968	-1.62060	-1.58832
PGK1_B	0.98981	0.85003	0.88879	0.65609	1.18845	1.06217	0.86183	0.72643
PHKA1_A	-0.36426	-0.34422	0.40239	0.62370	-0.02618	-0.08903	0.53164	0.43950
PHKA1_B	-1.39401	-1.44473	-0.78993	-0.73350	-1.41431	-1.24786	-0.14401	-0.43178
PHKA2_A	-1.51034	-1.38948	-0.30333	-0.37420	-0.66394	-0.79578	-0.00109	0.00446
PHKA2_B	-2.48232	-2.41487	-2.77780	-3.41309	-1.45387	-1.67410	-1.88883	-1.03194
PHKB_A	0.22610	0.34587	-0.38212	-0.26076	0.06558	0.06611	-0.57755	-1.25338
PHKB_B	0.66594	0.61746	0.73422	0.60324	0.11391	0.02638	0.21133	0.71505
PHKG1_A	-0.80934	-0.57327	-0.31928	-0.13909	0.86975	1.06063	0.32232	0.31850
PHKG1_B	0.95543	0.94226	0.34360	0.45306	0.67732	0.75805	0.75928	0.80309
PHKG2_A	0.30399	0.23629	0.98399	0.95661	0.18870	0.39658	0.63380	0.47896
PHKG2_B	0.74635	0.71309	0.34779	0.45188	-0.19446	-0.00502	0.33404	0.17032
PI4K2B_A	0.94086	0.67297	0.69820	0.65472	1.02893	0.87867	1.00232	0.90652
PI4K2B_B	-0.03282	-0.07749	0.50850	0.48247	0.92378	0.50084	0.90734	0.94536
PI4KII_A	-0.66647	-0.57092	-2.14523	-2.04440	0.36449	0.16008	-0.28974	-0.29568
PI4KII_B	-0.55828	-0.45008	0.27394	0.39866	0.41782	0.55092	0.23212	0.31639
PIK3AP1_A	-2.53960	-2.52615	-2.48296	-2.29178	-2.11575	-2.17769	-1.60295	-1.78094
PIK3AP1_B	-0.20027	-0.51305	0.26391	0.04511	0.44423	0.40323	-0.41593	-0.43473
PIK3C2A_A	0.98671	0.95952	0.52937	0.62756	0.56027	0.55796	0.41511	0.25635
PIK3C2A_B	1.39658	1.34876	1.01681	1.16086	1.33275	1.25245	1.22336	0.99494
PIK3C2B_A	-0.65006	-0.76675	0.31736	0.17907	-0.58024	-0.31359	-0.41474	-0.37026
PIK3C2B_B	1.38927	1.45641	1.10572	1.16946	1.05886	1.30739	0.98467	1.02758
PIK3C2G_A	1.03721	1.16241	1.04013	1.13535	1.03581	0.87999	0.44821	0.50505
PIK3C2G_B	1.54994	1.43979	1.08356	1.14052	1.08028	0.99963	0.84048	0.75327
PIK3C3_A	1.05507	1.11750	0.97506	0.99911	1.14836	1.03845	0.68708	0.61454
PIK3C3_B	1.18601	1.26622	1.10959	1.09173	1.21474	1.21961	0.66823	0.78923
PIK3CA_A	0.65586	0.58343	0.71412	0.58502	0.54484	0.53147	0.41859	0.75035
PIK3CA_B	1.51028	1.30629	1.03428	1.09778	1.33441	1.32712	1.05718	1.26927
PIK3CB_A	1.34620	1.27428	0.86553	0.69716	0.65318	0.54798	0.63939	0.68570
PIK3CB_B	1.49758	1.29115	1.13207	1.07063	1.00561	1.05436	1.13568	0.93361
PIK3CD_A	-0.99022	-0.72720	-1.36323	-1.83807	-1.63944	-1.60687	-0.54482	-0.52232
PIK3CD_B	1.32667	1.61608	0.95861	1.07825	1.40164	1.47879	1.24249	1.38076
PIK3CG_A	1.02528	0.91705	0.59216	0.63744	0.93232	0.88273	0.90271	1.05772
PIK3CG_B	1.42915	1.34474	1.11452	1.08327	1.44306	1.44022	1.11024	1.20913
PIK3R1_A	-1.83246	-1.92829	-1.02259	-0.68962	-1.52669	-1.61130	-1.14159	-1.23641
PIK3R1_B	1.25805	1.38812	1.09013	1.15997	1.32728	1.30685	1.05444	0.98998
PIK3R2_A	-0.56689	-0.60613	0.29872	0.19578	0.22992	0.30014	-0.31922	-0.24408
PIK3R2_B	1.44421	1.31835	0.74205	1.04968	1.11889	1.29305	0.98897	1.08223
PIK3R3_A	0.47925	0.35495	0.06583	0.28390	0.41410	0.40055	-0.63407	-0.80937
PIK3R3_B	0.43137	0.55178	-0.01176	0.16837	-0.26071	-0.05076	0.14418	0.14785
PIK3R4_A	-0.88898	-1.08080	-0.20291	0.00506	-0.44119	-0.74053	-1.46685	-1.45426
PIK3R4_B	1.01647	1.18803	-1.02382	0.80041	0.92325	0.90360	0.77919	0.67641
PIK4CB_A	-0.17130	-0.02885	0.36068	0.65524	0.20445	0.37171	-0.01187	-0.08233
PIK4CB_B	-0.78816	-0.77441	-1.01369	-0.86030	-0.50623	-0.80798	0.47439	0.35642
PIM1_A	-0.76468	-0.85285	1.06655	1.21287	1.11504	1.13008	1.16047	1.18049
PIM1_B	-0.33589	-0.46140	0.29963	0.70343	-0.31254	-0.28997	0.06121	0.15241
PIM2_A	0.95176	0.90047	0.84094	1.04906	0.68985	0.64298	0.54466	0.57106
PIM2_B	0.13294	0.13366	-0.76111	-0.63089	-0.93003	-0.89611	-0.47169	-0.48244
PINK1_A	-2.22619	-2.34586	-1.69435	-2.34184	-1.74844	-2.16523	-0.59500	-0.56025
PINK1_B	0.21374	0.02836	0.75981	0.49855	1.05476	1.02836	1.13986	1.07809
PIP5K1A_A	0.32930	0.64653	0.20522	0.81264	0.60713	0.33187	0.30686	0.26334
PIP5K1A_B	0.70555	0.69842	1.06577	1.08035	1.08870	1.06220	1.07226	0.95455
PIP5K1B_A	0.41850	0.43981	-0.45873	-0.46537	-0.88914	-0.90501	-1.01437	-0.84506
PIP5K1B_B	0.29238	0.27760	-0.30215	-0.59496	-0.81548	-0.90801	-0.96332	-1.26978
PIP5K1C_A	0.14773	0.26356	-0.34248	-0.58325	-1.10025	-1.27364	-0.74628	-0.91502
PIP5K1C_B	0.80039	0.88938	1.14005	1.07189	0.87672	0.81612	0.90331	0.82507
PIP5K2A_A	0.01219	-0.22675	0.76041	0.61025	0.07063	0.16659	0.47467	0.35758
PIP5K2A_B	0.49408	0.18317	0.67175	0.71017	0.87757	0.84244	0.70265	0.69248
PIP5K2B_A	0.63248	0.75839	0.18687	0.18500	0.84789	0.68049	0.48269	0.40401
PIP5K2B_B	0.99416	0.71620	0.13040	0.09225	-0.45821	-0.41021	0.21014	0.09456
PIP5K2C_A	0.48061	0.58652	0.72661	0.89315	0.27444	0.41849	0.14725	0.20470
PIP5K2C_B	0.83289	0.97853	0.53501	0.59156	0.86366	1.00417	0.37985	0.55931
PIP5K3_A	0.41652	0.54355	0.54153	0.59938	-0.10244	-0.05582	-0.10766	-0.31874
PIP5K3_B	0.91796	0.97886	0.97026	0.82233	0.51814	0.65760	0.47950	0.46193
PKE_A	-0.09606	-0.06996	0.05143	0.02637	-0.35697	-0.04434	0.16582	0.04323
PKE_B	-1.00440	-0.84557	-0.40190	-0.32824	-0.76676	-0.53826	-0.55901	-0.59686
PKIA_A	0.04846	-0.24540	0.40861	0.64590	0.79793	0.67117	0.54934	0.60112
PKIA_B	0.70489	0.60860	0.54958	0.73790	0.82278	0.71200	0.52459	0.42589
PKIB_A	-0.57019	-0.51115	-0.28521	-0.84984	-0.64132	-0.58063	-0.28910	-0.33664
PKIB_B	0.12779	0.31397	0.87478	0.36750	0.56391	0.44973	0.36342	0.42213
PKIG_A	1.45818	1.47028	0.97586	1.07115	1.17316	1.16914	1.07967	1.20227
PKIG_B	0.85330	0.75579	0.90643	1.02678	0.85388	0.79371	0.80718	0.76766
PKLR_A	-0.32891	-0.59536	-0.94953	-0.02149	-0.37149	-0.17396	0.57484	0.45754
PKLR_B	-0.35445	-0.10756	-0.15178	0.03524	-0.77305	-0.70264	-2.32892	-2.14102
PKM2_A	0.87065	0.91930	-0.08107	-0.03519	0.16427	0.03569	0.05748	0.01603
PKM2_B	0.45741	0.41447	-0.16393	-0.21923	0.37562	0.27903	0.80346	0.73565
PKMYT1_A	-0.50773	-0.19008	-0.84253	-0.83672	-1.44458	-1.46953	-0.80456	-0.54386
PKMYT1_B	-1.04593	-0.84166	-0.27229	-0.16918	0.04300	0.16761	-0.52448	-0.20627
PLK1_A	-2.82873	-2.93689	-3.78402	-3.67140	-6.82257	-7.20907	-8.06220	-7.94462
PLK1_B	-1.48186	-1.41395	-2.67816	-2.52681	-2.99526	-3.12422	-3.57271	-3.72238
PLK2_A	0.90329	1.03059	0.29840	0.23984	1.11504	1.11495	0.86783	0.84572
PLK2_B	-0.07603	0.02118	-0.19282	-0.59489	0.18613	-0.26028	0.21986	0.00113
PLK3_A	-0.61202	-0.31990	-0.79442	-0.70738	-0.38176	-0.23243	0.49210	0.65586
PLK3_B	-0.30314	0.10711	-1.39637	-1.06803	1.17550	1.20227	0.96937	1.09063
PLK4_A	0.44756	0.43437	0.94504	0.92451	1.18500	1.10392	1.23596	1.37540
PLK4_B	-0.40806	-0.54377	-1.44418	-1.45462	-0.99917	-0.86514	-0.26279	-0.11064
PMVK_A	0.26014	0.16094	0.44365	0.26055	-0.08276	-0.10914	0.18859	0.15797
PMVK_B	0.57672	0.49593	0.78713	0.55975	0.73138	0.83154	0.88123	0.90943
PNCK_A	-0.15818	-0.12633	0.92244	0.87179	0.53633	0.56280	0.54370	0.62842
PNCK_B	-0.04277	-0.21823	0.13556	0.08050	0.27162	0.36220	0.36970	0.37433
PNKP_A	-1.04123	-1.08683	-1.93689	-1.89093	-0.85237	-0.85761	-0.62126	-0.59279
PNKP_B	0.94971	1.07527	0.41058	0.53977	0.72740	0.78133	0.75547	0.75167
PRKAA1_A	1.06620	0.93441	-0.02010	0.02818	-0.19240	-0.05145	-0.54173	-0.57546
PRKAA1_B	-0.31199	-0.42183	-0.80105	-0.67760	-0.65296	0.01944	-0.64106	-0.77149

Additional File 2: Z-score values for kinase siRNA library screen of Ewing's sarcoma cells (cont)

siRNA	TC-32_a	TC-32_b	TC-71_a	TC-71_b	SK-ES-1_a	SK-ES-1_b	RD-ES_a	RD-ES_b
PRKAB1_A	0.51283	0.51348	-1.81940	-1.95920	-0.57717	-0.36568	-0.16123	-0.29014
PRKAB1_B	-1.12395	-1.06100	-1.64826	-1.55789	-3.15363	-3.39971	-1.32731	-1.48331
PRKAB2_A	-0.08419	0.41056	0.18436	0.18570	-0.74504	-0.45279	-0.18954	-0.03600
PRKAB2_B	1.45229	1.59555	1.06600	1.16265	0.86641	1.10278	1.05112	1.15145
PRKACA_A	0.54492	0.54305	0.70761	0.71311	0.50422	0.58411	0.71257	0.56501
PRKACA_B	0.26764	0.08718	-0.48162	-0.49956	-0.78249	-0.89128	-0.23231	-0.59136
PRKACB_A	-0.10557	-0.29482	-0.84389	-0.87859	-1.11117	-1.04675	-0.15629	-0.42549
PRKACB_B	-0.26625	-0.44398	-0.09198	-0.07433	-1.01303	-1.08970	-0.29073	-0.22521
PRKACG_A	-0.40842	-0.31388	-1.10810	-1.17854	-0.18569	-0.22993	-0.17278	-0.30662
PRKACG_B	-0.17752	-0.51529	-0.71778	-0.90824	-0.35502	-0.21804	-0.34914	-0.43554
PRKAG1_A	-0.93465	-0.94974	0.11219	0.25613	0.61789	0.61227	0.18508	0.26912
PRKAG1_B	0.32968	0.58858	-0.70688	-0.95170	0.30525	0.33466	0.81719	1.02570
PRKAG2_A	0.74236	0.61338	0.39589	0.62526	0.12535	-0.00304	0.17561	0.05606
PRKAG2_B	0.04160	-0.32871	0.69146	0.53999	0.12796	0.06962	0.18023	0.31914
PRKAG3_A	-0.80102	-1.03565	-0.84296	-0.61942	-1.77397	-1.56212	-0.99694	-1.01012
PRKAG3_B	0.27609	0.14403	-0.77409	-0.54157	-1.31899	-1.11646	-0.42599	-0.50494
PRKAR1A_A	0.58690	0.29201	-0.16265	-0.14310	-0.65898	-0.51973	0.15082	0.23594
PRKAR1A_B	1.39894	1.13006	0.80560	0.87054	0.67743	0.73856	0.86505	0.83760
PRKAR2A_A	1.05623	1.08477	1.26317	1.06759	1.43448	1.34929	1.20367	1.11956
PRKAR2A_B	-1.90247	-2.22404	-1.86355	-1.92566	-1.07419	-1.17078	-1.15984	-1.27028
PRKAR2B_A	0.69508	0.59151	-0.08833	0.05987	-0.94644	-0.98294	-0.72738	-0.72059
PRKAR2B_B	-0.93534	-0.99369	-0.53163	-0.93819	-0.38199	-0.43883	0.83992	0.89521
PRKCA_A	-0.17346	-0.57366	-1.15085	-0.60819	-1.25296	-1.29719	-0.84220	-0.78962
PRKCA_B	-2.68409	-2.70459	-4.59873	-4.63488	-3.38698	-3.43753	-4.30543	-4.13332
PRKCABP_A	1.15783	1.20812	1.01794	1.22567	1.06387	1.06723	0.81658	0.95969
PRKCABP_B	-0.19253	-0.00072	-0.21026	-0.43128	-0.21683	-0.27999	-1.22724	-1.17385
PRKCB1_A	0.02519	0.14401	-0.03815	0.10856	-0.18001	-0.14762	0.35774	0.26045
PRKCB1_B	0.62818	0.53298	1.12748	1.04211	1.22877	1.42362	1.05709	1.12797
PRKCBP1_A	0.93611	0.86928	0.63031	0.64623	0.99492	0.88595	1.07145	1.09799
PRKCBP1_B	0.17024	0.66873	0.66317	0.71958	0.98864	0.85040	0.41146	0.62909
PRKCD_A	0.39689	0.48747	0.01256	0.19200	-0.04155	0.03935	-0.56569	-0.70503
PRKCD_B	-0.38318	-0.13782	-1.85033	-1.35945	-0.73218	-0.29181	0.30640	0.25318
PRKCDBP_A	-0.33663	-0.01621	-0.53842	-0.81366	-0.69671	-0.76890	-0.37519	-0.52979
PRKCDBP_B	0.14175	0.29636	0.03633	-0.05734	0.43868	0.54205	-0.20820	-0.06237
PRKCE_A	0.91760	0.74858	0.85922	0.70405	0.32641	0.38128	0.56899	0.44446
PRKCE_B	-1.71293	-1.50397	-0.89519	-0.91760	-0.64684	-0.71832	-0.19262	-0.21818
PRKCH_A	0.70291	0.37533	0.53797	0.62836	-0.04507	-0.06656	-0.14659	-0.12306
PRKCH_B	-0.36642	-0.29705	0.90453	0.81117	-0.06015	0.06825	-0.17070	-0.13863
PRKCI_A	1.47207	1.23830	1.10454	1.00336	1.09290	1.13936	1.07953	1.06694
PRKCI_B	-0.60420	-0.16786	-0.09816	-0.06836	-0.57000	-0.22220	-0.67798	-0.74247
PRKCL1_A	-0.62484	-0.42377	-2.67579	-2.61435	-1.55672	-1.74940	-1.48516	-1.34042
PRKCL1_B	-1.06079	-0.56383	-2.67254	-2.48017	-2.65126	-2.56552	-1.24224	-1.33888
PRKCL2_A	1.26605	1.23103	1.08218	1.11305	0.87076	0.98879	0.93938	0.91250
PRKCL2_B	-0.76085	-0.66835	-0.46723	-0.27443	-0.79341	-0.69958	-0.17355	0.02534
PRKCN_A	0.30619	0.47106	-0.11509	-0.32610	0.09974	0.17355	0.98951	0.89401
PRKCN_B	-1.51825	-1.44994	0.08523	0.06956	-1.18698	-1.14362	-0.17878	-0.12867
PRKCQ_A	0.78557	0.83265	0.85572	0.72705	0.92932	1.01098	1.00273	1.10484
PRKCQ_B	0.19094	-0.36682	-1.95553	-1.66238	-1.05679	-0.90371	-1.31618	-1.42120
PRKCZ_A	0.34518	0.13164	0.92219	0.74398	0.83400	1.00583	0.85586	0.80193
PRKCZ_B	1.06027	1.07260	0.99597	1.06224	0.96378	1.19545	0.97871	0.98288
PRKD2_A	-1.05545	-1.10774	-1.60732	-1.37328	-1.57299	-1.52924	-0.07458	0.17158
PRKD2_B	0.53986	0.42329	0.51758	0.73872	-0.30628	-0.01261	-0.37305	-0.21301
PRKDC_A	0.55064	0.57082	0.87784	0.90475	0.95802	0.96804	0.69775	0.53177
PRKDC_B	-0.23935	-0.08692	-0.40893	-0.61027	0.39754	0.24541	-0.00370	-0.56886
PRKG2_A	1.02782	1.10607	0.32353	0.44764	0.40527	0.65602	0.65922	0.75392
PRKG2_B	1.05335	1.29244	0.88890	1.04088	0.71344	0.83271	0.55719	0.68076
PRKRA_A	1.30475	1.30107	1.29779	1.13400	1.18700	1.24194	1.26797	1.24170
PRKRA_B	0.18742	0.03755	0.97326	0.63496	0.30869	0.36181	0.84233	0.83890
PRKRIR_A	0.17730	-0.27981	0.37607	0.38951	1.18149	1.02295	0.48333	0.44534
PRKRIR_B	0.52288	0.40178	0.87264	0.77441	1.05182	0.68514	0.68105	0.62564
PRKX_A	0.36318	0.26133	0.23221	0.22664	0.15834	0.13280	0.36991	0.40728
PRKX_B	1.15089	1.02033	0.85436	0.88774	1.02218	1.05409	1.06050	0.83337
PRKY_A	1.12180	0.83793	1.00433	0.93978	0.52151	0.59623	0.42591	0.47703
PRKY_B	-1.56214	-1.94829	0.02201	-3.27445	-0.62896	-0.68103	-0.04749	-0.19847
PRPF4B_A	-0.28675	-0.56100	0.13235	0.15204	-1.19462	-1.19714	-0.98111	-1.01470
PRPF4B_B	-0.04263	0.21213	0.53850	0.16061	0.26272	0.15330	0.29165	0.28620
PSKH1_A	0.01916	0.02718	0.13351	0.12953	0.98061	1.05889	0.89442	0.88538
PSKH1_B	-0.14327	-0.05646	-0.05745	0.12446	-0.50531	-0.46217	0.12618	0.12384
PSKH2_A	0.66320	0.38044	0.47683	0.62752	0.71181	0.53194	0.48983	0.69042
PSKH2_B	-0.04394	-0.50349	-0.31902	-0.37877	-0.58817	-0.98479	0.14801	0.28375
PTK2_A	-1.13109	-1.08352	-1.42714	-1.20220	-0.51087	-0.53139	-0.33563	-0.28804
PTK2_B	0.58155	0.76855	0.90876	0.83979	0.81625	0.92420	0.82064	0.77488
PTK2B_A	0.84573	0.80759	0.94462	0.78025	0.58352	0.60596	0.88586	0.90075
PTK2B_B	0.68862	0.67715	0.21614	0.05974	1.07167	0.95090	0.68201	0.60156
PTK7_A	0.00895	0.01731	0.43593	0.51303	-0.09148	0.02064	-0.14758	-0.28615
PTK7_B	-2.04957	-2.15161	-1.01561	-0.93330	-1.51544	-1.60549	-2.68865	-2.86326
PTK9_A	-0.43753	-0.44893	0.56246	0.18284	-0.49814	0.00635	-0.12690	-0.24772
PTK9_B	-1.48290	-1.38247	-4.17033	-4.21569	-2.12681	-2.02644	-1.33449	-1.83377
PTK9L_A	-0.22637	-0.10211	-0.57235	-0.63634	-0.50322	-0.34495	0.17236	0.13891
PTK9L_B	-0.77904	-0.46558	-2.13071	-2.20165	-0.57765	-0.50109	0.03309	-0.14344
PXK_A	-2.24243	-2.21577	-3.75988	-3.72273	-2.23635	-2.14898	-3.16595	-3.18361
PXK_B	0.91541	0.78011	0.70759	0.57356	0.35764	0.38233	0.45632	0.57730
RAF1_A	1.39738	1.33926	1.19588	1.12863	1.29520	1.41005	0.89652	0.98477
RAF1_B	0.35874	0.37848	0.73170	0.63192	0.35250	0.50292	-0.07650	-0.00703
RAGE_A	-3.15949	-2.99751	-0.15511	-0.33365	-1.72405	-1.60666	-1.48329	-2.10744
RAGE_B	-1.50334	-1.30705	-1.09054	-2.34526	-0.69995	-0.82736	0.17432	-1.44275
RBKS_A	0.97606	1.08546	0.91769	0.81705	0.55603	0.36787	0.48799	0.44531
RBKS_B	-0.54828	-0.31142	0.77925	0.73383	0.62832	0.54740	0.11669	0.34038
RET_A	0.24923	0.07902	0.43809	0.39242	0.44922	0.53436	0.62687	0.49514
RET_B	0.55848	0.47707	0.70308	0.73916	0.37214	0.51025	0.48097	0.34901
RIOK1_A	0.70326	0.43327	0.49792	0.45205	0.12107	0.80198	0.49769	0.47372
RIOK1_B	-0.71296	-1.15266	-0.51995	-0.64562	0.33678	0.44995	0.54027	0.49162
RIOK2_A	0.06659	0.18983	-0.03406	0.05830	-0.79459	-0.70090	0.24544	0.23721
RIOK2_B	-0.92571	-0.47917	-0.11196	0.04996	-0.96897	-0.81022	-0.52024	-0.75865
RIOK3_A	0.66761	-1.28143	0.54019	0.53835	0.48546	0.80375	1.35870	1.33687
RIOK3_B	0.12797	0.35205	-0.97952	-1.25566	-1.21728	-0.41962	-0.11912	-0.25342
RIPK1_A	1.48012	1.32740	1.18844	0.98420	1.59139	1.46664	1.45665	1.41098
RIPK1_B	0.40658	0.42953	-0.30084	-0.29206	0.36512	0.15796	0.82322	0.94253
RIPK2_A	0.63004	0.69427	0.50976	0.23868	0.54024	0.76965	0.74183	0.85896
RIPK2_B	1.25397	1.40151	0.92709	1.03742	1.25711	1.26585	0.99738	1.06327
ROCK1_A	0.08622	-0.08202	0.53850	0.19714	0.17970	0.32255	0.22308	0.20941
ROCK1_B	-0.06934	-0.34332	0.28961	0.41737	-0.28152	-0.12162	-0.53567	-0.45084
ROCK2_A	-0.06206	-0.29955	-0.23360	-0.33305	-1.19834	-1.42422	-0.79668	-0.83327
ROCK2_B	0.32388	0.17471	-0.17966	0.10318	0.26883	0.18081	0.79011	0.82077
ROR1_A	-0.04394	0.19195	0.12880	0.07793	0.33518	0.12564	-0.40513	-0.29167
ROR1_B	0.02983	-0.09466	0.19508	0.24134	0.09721	0.03454	-0.03064	0.07088
ROR2_A	-1.32935	-1.47466	-0.03018	0.21033	0.07050	0.23280	0.50693	0.46005
ROR2_B	-1.60932	-1.67669	-0.93919	-0.91986	-0.83962	-0.70747	-0.33329	-0.43859
ROS1_A	0.92386	0.92332	1.04168	1.13468	1.07890	0.97321	0.84865	1.14176
ROS1_B	1.21339	1.17531	0.86962	0.99457	0.56036	0.37554	0.86162	0.94849
RPS6KA1_A	0.59748	0.71468	0.31531	0.61122	1.26640	1.18922	0.91731	1.15698
RPS6KA1_B	-0.32396	-0.02907	0.08583	0.08149	-0.04774	-0.23944	-0.09422	0.00479
RPS6KA2_A	0.59414	0.59698	-0.44583	-0.21803	0.24503	0.45795	0.66843	0.75000
RPS6KA2_B	0.21317	0.06577	0.57718	0.65314	0.40708	0.62340	0.03908	0.17001
RPS6KA3_A	0.49373	0.46529	0.94415	0.96135	0.67906	0.71863	0.49003	0.53557
RPS6KA3_B	0.29863	0.19617	0.46913	0.56835	-0.08853	0.14339	-0.11917	-0.32617
RPS6KA4_A	-0.21092	-0.24700	0.22694	-0.19115	-0.59836	-0.49578	-0.17427	-0.71877
RPS6KA4_B	0.52413	0.14143	0.40818	0.69146	0.48250	0.54187	0.40778	0.43761
RPS6KA5_A	0.06304	0.08719	-1.09997	-0.87197	-1.24576	-1.09141	-0.47753	-0.58128
RPS6KA5_B	0.41135	0.50077	0.56836	0.73706	0.58775	0.45244	0.31226	0.43915
RPS6KA6_A	-0.18420	-0.20233	-0.41821	-0.37017	-1.59453	-1.47364	-1.01929	-1.05249
RPS6KA6_B	1.22298	1.05722	0.99285	0.98067	0.57524	0.46773	0.26189	0.32408
RPS6KB2_A	-1.27733	-0.97371	0.20973	0.12673	-0.35421	-0.09144	-0.80190	-0.83303
RPS6KB2_B	-1.02058	-0.90471	-1.46084	-1.03190	-1.46253	-1.42147	-0.71410	-1.00683
RPS6KC1_A	0.78378	0.99163	0.87742	1.00741	0.36603	0.68761	0.30347	0.66709
RPS6KC1_B	-0.94969	-0.91056	-2.30611	-2.14083	-1.58221	-1.50204	-0.44665	-0.74099
RPS6KL1_A	-2.71717	-2.71971	-0.49063	-0.46766	-0.55892	-0.46534	-0.78168	-0.97110
RPS6KL1_B	-1.72040	-1.69327	-2.12139	-1.76906	-4.46118	-4.21207	-3.76879	-3.23123
RYK_A	0.38192	0.24948	0.90164	0.85428	0.80604	0.72666	0.82609	0.95604
RYK_B	-1.05434	-0.89957	-0.80960	-1.11443	-1.01782	-0.84164	-3.13902	-3.10130
SGK_A	-0.01640	0.08654	0.33949	0.46081	-0.41265	-0.25455	-0.69123	-0.82880
SGK_B	0.74767	0.68693	0.56357	0.74203	-0.44766	-0.27145	0.25705	0.50569
SGK2_A	-1.70517	-1.45685	0.69051	0.71967	-0.50595	-0.29826	-0.40920	-0.23469
SGK2_B	-0.47623	-0.34481	-0.11172	-0.05359	-1.26264	-0.90525	-1.39552	-1.15602
SGKL_A	0.22255	0.44054	0.77201	0.86209	0.74848	0.76861	0.68076	0.62768
SGKL_B	0.86287	1.11127	0.87110	0.96959	0.81868	0.97455	0.77863	0.71612
SH3KBP1_A	0.96839	1.00681	0.71209	0.97144	0.81287	0.55325	0.67475	0.46067
SH3KBP1_B	0.74836	0.83444	0.88936	0.71505	1.25245	1.21946	0.91643	0.80778
SKP2_A	-1.94658	-2.16800	0.05713	0.29477	0.07551	0.06764	0.10118	0.21188
SKP2_B	0.53961	0.76101	0.02170	0.19128	0.39456	0.39222	-0.32241	-0.18413
SLK_A	0.74542	0.61138	0.51951	0.23789	0.86745	0.69508	0.40947	0.40800
SLK_B	-0.91328	-0.86634	-3.66640	-3.62982	-2.46937	-2.57620	-2.23739	-2.26540
SMG1_A	0.23162	0.22652	0.18892	0.03362	-0.03409	-0.04437	-0.03987	0.12250
SMG1_B	0.99915	0.90117	0.61426	0.39078	1.06518	1.16464	0.74244	0.83348
SNARK_A	0.47164	0.37174	0.89179	1.20473	0.31444	0.37253	0.69899	0.57625
SNARK_B	0.61448	0.71599	0.54061	0.85062	0.45144	0.52648	0.51756	0.61241
SNF1LK_A	-1.43810	-1.76340	-0.78159	-0.77293	-1.67178	-1.62214	-1.37561	-1.74049
SNF1LK_B	0.42124	0.67637	0.47517	0.63587	0.49807	0.54820	0.22247	0.13879
SNF1LK2_A	0.98484	0.97727	1.05583	0.92753	0.68991	0.41879	0.72623	0.86901
SNF1LK2_B	1.07738	0.96287	1.01489	1.04989	0.91250	0.78449	0.72824	1.11754
SNRK_A	0.01753	-0.05729	0.20139	0.31834	-0.42752	-0.46154	0.05656	-0.04763
SNRK_B	0.78807	0.60533	0.51976	0.65732	0.22406	0.22098	0.13845	0.29216
SPEC2_A	0.05135	-0.03875	0.07706	-0.11121	-0.97631	-0.75768	-0.36697	-0.07892
SPEC2_B	-0.02268	0.20093	-0.53598	-0.13419	-1.06925	-0.96388	-0.43034	-0.36257
SPHK1_A	0.22684	0.33446	0.20041	-0.05802	-0.07344	0.02728	-0.36088	-0.48985
SPHK1_B	-1.33993	-0.82808	0.15425	0.30492	-0.28293	-0.40903	-0.68758	-0.72368
SPHK2_A	-1.08001	-0.83378	-0.47000	-0.46024	0.22867	0.13262	-0.93576	-0.64722
SPHK2_B	0.70805	0.85292	0.45531	0.42373	0.43905	0.28389	0.74289	0.63257
SRC_A	0.92437	1.03174	1.03306	1.11121	0.98677	1.07271	0.67376	0.75350
SRC_B	0.19238	0.09931	0.36831	0.42364	-0.57654	-0.58069	-0.09868	-0.02947
SRPK1_A	-1.36746	-1.25320	0.54767	0.41430	0.64719	0.64937	0.36151	0.18782
SRPK1_B	0.39781	0.63072	-0.72129	-0.89702	0.19494	0.26770	0.36596	0.36704
SRPK2_A	-0.40800	-0.51962	-0.09879	-0.19192	-0.29726	-0.54699	0.34114	0.25024
SRPK2_B	-0.12506	0.10724	-0.31838	-0.08800	0.36709	0.06554	0.07193	-0.29209
STK10_A	-3.07818	-2.95373	-2.49782	-3.39999	-4.22718	-3.94374	-5.71332	-5.44365
STK10_B	-1.93917	-2.22199	-3.70357	-4.08891	-3.13732	-3.10722	-3.20617	-2.31626
STK11_A	0.73580	0.88750	0.48470	0.39891	0.38398	0.54619	-0.03173	-0.13904
STK11_B	-1.84234	-1.59514	-2.42227	-1.99353	-2.43087	-2.23636	-1.99493	-2.08032
STK11IP_A	0.30762	0.40162	0.06457	0.13942	0.64581	0.67290	0.72778	0.82165
STK11IP_B	-2.41098	-2.34959	-1.16729	-1.15217	-0.17967	-0.09968	-0.23759	-0.11197
STK16_A	-0.23316	-0.04369	-0.36316	-0.22463	0.18882	0.27344	-1.06124	-1.00041
STK16_B	-1.58413	-1.58995	-0.45804	-0.35837	-0.80814	-0.73681	-2.44878	-2.92131
STK17A_A	0.87621	0.79872	1.07425	1.04987	0.84768	0.61770	0.53554	0.81843
STK17A_B	0.49955	0.65831	0.87692	0.82017	-0.54924	-0.88033	-0.16429	-0.15477
STK17B_A	0.69441	0.63399	0.95288	0.80123	0.58172	0.80317	0.77302	0.70415
STK17B_B	0.38178	0.22663	0.63177	0.56969	0.30552	0.41475	1.19307	1.09545
STK19_A	0.85743	0.74603	0.57867	0.56932	0.57288	0.28995	0.52286	0.73740
STK19_B	-0.00110	0.00490	0.37266	0.23538	0.14146	0.08690	0.26717	0.17640
STK22B_A	0.76105	0.77855	0.88657	0.86495	0.27101	0.32612	0.47363	0.60346
STK22B_B	0.73898	0.50660	-0.22414	-0.43507	0.12709	0.17850	0.43697	0.62244
STK22D_A	-0.06740	-0.06552	-0.28679	0.15433	0.00560	-0.10003	0.00461	0.09027
STK22D_B	0.17947	0.38032	-0.10633	0.05123	0.27374	0.23981	0.20239	0.22709
STK24_A	-0.91734	-0.81496	-0.18581	-0.10982	0.52519	0.37584	-0.18752	-0.08607
STK24_B	1.39753	1.33244	1.04039	1.19227	1.21458	1.21799	1.07213	1.09827
STK25_A	0.39411	0.33720	-0.16671	-0.34358	-0.88798	-0.96062	-1.30857	-1.25482
STK25_B	-1.92849	-1.89711	-0.95122	-0.87856	-0.23362	-0.11536	-2.32227	-2.30242
STK29_A	-0.64402	-0.92872	-0.20469	0.00343	-0.90996	-0.89684	-0.96327	-0.89021
STK29_B	-0.20849	-0.50374	-0.67716	-0.46446	-0.62848	-0.93498	-0.69433	-0.74861
STK3_A	-0.11792	-0.00630	-0.88921	-0.46223	-0.97299	-1.12972	0.52727	0.64414
STK3_B	0.25523	0.29737	-0.07232	-0.18895	-0.02426	0.04413	0.32246	0.43011
STK31_A	-0.85354	-1.15794	-0.55444	-0.86618	0.17453	0.00164	0.40789	0.66726
STK31_B	-0.99693	-0.16122	-0.44251	-0.42061	-2.47021	-3.02391	-1.24361	-1.15099
STK32B_A	0.13455	0.17246	0.19897	0.01815	0.08586	-0.04131	0.03646	-0.15747
STK32B_B	-1.64470	-1.51579	-0.03487	0.13945	0.02797	-0.21297	-0.96260	-0.78880
STK33_A	-0.79414	-1.02087	0.19221	0.34500	0.74338	0.97121	0.47690	0.53264
STK33_B	-1.80599	-1.83750	0.79242	1.13264	-0.92734	-0.94623	-0.56891	-1.12140
STK38_A	0.59451	0.41825	0.85335	0.83253	1.08995	0.91321	0.48330	0.58461
STK38_B	0.58521	0.30200	0.15934	0.19981	-0.18589	-0.18119	-0.27644	-0.40479
STK38L_A	1.14749	1.25391	0.84869	0.18756	0.97339	1.01672	0.59475	0.66358
STK38L_B	-3.01085	-2.66508	-1.27015	-1.17446	-2.52095	-2.58170	-2.05888	-2.20444
STK39_A	0.16505	0.15171	0.52794	0.51653	0.69784	0.72784	0.46457	0.63229
STK39_B	-0.34410	-0.37910	0.04437	-0.15464	0.15171	-0.02367	-0.18602	-0.24681
STK4_A	-0.81796	-0.78849	-0.01562	-0.12603	-0.98650	-1.16400	-0.50375	-0.38784
STK4_B	1.11632	1.07315	0.97791	1.05560	1.12236	1.14547	1.17034	1.15304
STK6_A	-0.41693	-0.66465	-0.44499	-0.28228	-0.75643	-0.06043	-0.82152	-1.18862
STK6_B	-1.92391	-2.07811	-1.02725	-0.94840	-0.73656	-0.82823	-0.57681	-0.85509
SYK_A	0.40130	0.32842	0.68755	0.76344	0.84426	0.81998	1.12248	1.08005
SYK_B	-1.06010	-1.11338	0.49991	0.37999	0.37469	0.37038	0.82321	0.89095
TAO1_A	0.74410	0.71311	0.52105	0.60784	1.08005	1.35615	0.84154	0.86866
TAO1_B	0.01813	-0.00633	0.42835	0.36398	0.19021	0.17756	0.94248	0.86088
TAOK3_A	0.02923	-0.05135	-0.32999	-0.23469	0.18612	0.03365	0.68703	0.73672
TAOK3_B	-2.43685	-2.47137	-2.50659	-2.82278	-3.01515	-3.01725	-3.55013	-3.75659

Additional File 2: Z-score values for kinase siRNA library screen of Ewing's sarcoma cells (cont)

siRNA	TC-32_a	TC-32_b	TC-71_a	TC-71_b	SK-ES-1_a	SK-ES-1_b	RD-ES_a	RD-ES_b
TBK1_A	-0.38917	-0.37902	0.72730	0.79508	-0.70343	-0.58038	0.16441	0.15303
TBK1_B	0.72731	0.88761	0.99230	1.11196	1.17098	1.18047	1.04720	0.89210
TEC_A	0.60531	0.70916	0.50766	0.78818	0.80114	0.93412	0.58075	0.73027
TEC_B	0.07848	-0.01292	-0.56495	-0.63293	0.43622	0.68843	0.50105	0.58876
TEK_A	1.03892	1.14032	1.09116	1.00605	1.19961	1.23048	1.03514	1.20181
TEK_B	0.11671	0.45095	0.95532	0.67979	0.75848	0.88764	0.54064	0.51901
TESK1_A	1.36376	1.48123	1.17500	1.07476	1.42639	1.30893	0.84996	0.93785
TESK1_B	0.29520	0.37667	-0.21080	-0.21476	0.46010	0.21993	-0.01575	-0.01112
TESK2_A	1.23806	0.79946	0.88601	1.02259	0.91594	0.75897	0.80110	0.69458
TESK2_B	-1.04812	-1.26237	0.01158	0.00294	-1.16185	-1.10944	-0.82450	-0.73271
TGFBR1_A	1.26753	1.41133	1.33468	0.79157	1.58073	1.44541	1.24785	1.31335
TGFBR1_B	0.38295	0.53091	0.06202	0.08500	1.06986	0.93996	0.78677	0.71417
TGFBR2_A	1.44904	1.41578	1.15817	1.09973	1.32192	1.29946	1.10243	1.18306
TGFBR2_B	-1.40712	-0.91559	-1.79059	-2.85801	-1.68037	-1.97780	-2.45560	-2.18375
TK1_A	0.65995	0.83036	-0.06946	0.25267	0.62814	0.65739	0.47960	0.63097
TK1_B	-2.61750	-1.63656	-0.59023	-0.37114	-1.91392	-1.77207	-2.79068	-2.79438
TK2_A	-3.73204	-3.31600	-1.07047	-1.22204	-1.76741	-2.19718	-0.70923	-0.35839
TK2_B	-0.37063	-0.23426	-1.65454	-1.39653	0.23526	0.24206	0.26374	0.28924
TLK1_A	0.72665	0.74381	0.06271	-0.13400	0.10849	0.46341	0.36114	0.23402
TLK1_B	0.84296	0.86166	0.13589	-0.01513	0.23614	0.23545	-0.05582	-0.03095
TNIK_A	0.78239	0.64295	0.33300	0.27542	0.43669	0.41707	0.48063	0.54384
TNIK_B	-0.04980	-0.02722	-1.77583	-2.17841	-1.57213	-1.47000	-1.11717	-1.21588
TNK1_A	-0.31541	-0.22667	0.43332	0.39894	-0.17370	0.04429	0.37559	0.50176
TNK1_B	-0.97217	-1.08471	-1.19302	-1.14548	-2.23816	-1.85695	-1.15551	-1.13821
TNK2_A	-2.44889	-2.55809	-1.80730	-1.64313	-2.26008	-2.17356	-2.05721	-2.02896
TNK2_B	-1.50292	-1.78361	-2.12661	-1.98065	-1.52617	-1.50663	-1.89872	-2.54192
TNNI3K_A	0.75738	1.08991	1.23495	1.36891	0.90882	1.00995	0.77436	0.67682
TNNI3K_B	-1.72508	-1.66169	-1.01074	-0.64054	-0.46710	-0.26771	0.05086	0.07822
TPK1_A	-0.03321	-0.27614	-0.59675	-0.58554	-0.94786	-1.26754	-0.44637	-0.39649
TPK1_B	-0.03123	-0.04296	-0.39272	-0.35328	0.34351	-0.13587	-0.01319	-0.17248
TRAD_A	1.02416	1.09977	0.62086	0.73282	0.45478	0.52392	0.34631	0.16073
TRAD_B	0.86299	0.78642	1.05353	1.09825	0.34057	0.41807	0.44918	0.55354
TSKS_A	-0.68314	-0.79367	-2.42791	-1.12529	-1.51606	-1.58299	-1.48336	-1.48175
TSKS_B	-0.13159	-0.15846	-0.05992	-0.09114	0.04731	0.15825	0.10588	0.21225
TTBK1_A	0.96648	1.03264	0.54592	0.78584	0.61255	0.64349	0.91713	0.69448
TTBK1_B	0.67206	0.73362	0.82626	0.98367	0.64809	0.73551	0.76318	0.70024
TTBK2_A	-0.57411	-0.26567	0.52358	0.48025	0.87961	0.79130	0.60381	0.52000
TTBK2_B	0.88310	1.03686	0.81566	0.84413	0.20269	0.00654	0.30223	0.30400
TTK_A	-0.24602	0.07578	-0.12884	-0.24941	-0.56782	-0.41384	0.09244	0.21054
TTK_B	1.26094	1.31798	1.17475	1.10223	1.38859	1.43626	1.21221	1.44752
TYK2_A	0.85958	0.84526	0.46329	0.54037	0.38352	0.44316	0.69832	0.50437
TYK2_B	0.76552	0.56727	-0.06234	-0.06403	0.24806	0.12562	0.08330	0.14753
TYRO3_A	-0.95042	-0.87209	-1.76768	-1.68097	-1.44958	-1.20369	-1.70159	-1.75167
TYRO3_B	0.47898	0.53677	0.55032	0.35887	0.49270	0.71848	-0.28198	-0.44094
UCK1_A	-1.44152	-1.21318	-1.19733	-1.14125	-1.31263	-1.44440	-0.71204	-0.96027
UCK1_B	-1.85893	-1.57671	-1.34059	-1.73710	-1.20544	-1.43812	-1.88856	-2.13579
UGP2_A	1.18066	1.31497	0.91231	0.70900	0.97678	1.06256	0.85794	0.73861
UGP2_B	0.34071	0.18675	-0.16101	-0.27995	-0.16552	0.04602	-0.00421	0.04433
ULK1_A	0.02642	-0.00329	-0.45458	-0.61250	-2.05145	-2.03096	-0.67577	-0.64991
ULK1_B	0.00946	-0.04053	1.15045	1.28431	0.31886	0.41880	0.16674	0.35751
ULK2_A	0.10851	-0.14602	-0.68339	-0.61043	-1.54581	-1.62524	-0.81032	-0.97249
ULK2_B	-1.32086	-1.04877	-0.33240	-0.26238	-0.26604	0.13201	0.15211	0.18735
UMP-CMP_A	-0.51721	-0.29135	0.20966	0.31712	-0.03139	-0.11836	0.46861	0.45104
UMP-CMP_B	-0.17662	-0.07479	0.21383	0.41226	-0.98839	-1.04957	-0.58945	-0.39414
UMPK_A	1.22295	1.22386	0.91841	1.16854	1.27551	1.32474	1.03861	1.15583
UMPK_B	-0.88720	-0.75017	-0.36683	-0.19886	-1.21660	-1.28749	-1.11050	-1.19310
URKL1_A	-0.70301	-0.53523	0.09497	0.06383	-1.51675	-1.61817	-0.49611	-0.53967
URKL1_B	0.78264	0.78569	0.65268	0.57310	0.11743	0.23722	0.34981	0.38755
VRK1_A	1.27797	1.26348	0.64938	0.26682	0.51507	1.12572	0.59997	0.66362
VRK1_B	1.47578	1.55928	1.00136	1.00394	0.81961	0.59557	0.97161	0.99177
VRK2_A	1.07123	0.73354	0.28500	0.21308	0.49483	0.43402	0.41770	0.42639
VRK2_B	1.17138	1.13272	0.73209	0.61990	0.45504	0.72079	0.87989	0.79574
VRK3_A	-0.62447	-0.46915	-0.71277	-0.80662	-0.51506	-0.59435	-0.77920	-0.99130
VRK3_B	0.42274	0.57037	-1.09015	-0.94686	0.54787	0.49974	0.05643	-0.20490
WNK1_A	1.50862	1.23819	1.09125	1.18413	1.32629	1.33563	1.27068	1.09278
WNK1_B	0.34106	-0.00237	0.21094	0.18993	0.77942	0.63167	1.19306	0.96318
WNK3_A	0.90576	0.82852	0.57351	0.48872	1.10336	1.07008	0.64885	0.63672
WNK3_B	0.77653	0.71518	0.79130	0.80358	0.96950	0.83212	0.33590	0.25951
WNK4_A	-0.12310	-0.27231	-0.08358	-0.12139	-0.70540	-0.53908	-0.75417	-0.72373
WNK4_B	-2.05354	-2.30386	0.04115	-0.02268	0.89880	0.97059	0.32768	0.20846
XYLB_A	1.59484	1.38478	0.83475	0.97715	1.29051	1.12568	1.16549	0.99902
XYLB_B	0.55768	0.58559	0.62083	0.45708	0.45825	0.20079	1.12203	1.08883
YES1_A	0.33046	0.29023	0.67904	0.69747	1.01271	0.84129	0.21321	0.27608
YES1_B	-0.84846	-0.84879	0.70788	0.71962	0.41409	0.14251	-0.08586	-0.07541
ZAK_A	0.83247	0.37254	-0.18219	-0.14222	-1.62640	-1.62933	-0.30195	-0.50048
ZAK_B	1.24950	1.27568	0.34021	0.76111	0.63730	0.85090	0.73959	0.55250
